# New Insights into Intersystem Crossing in Substituted Aromatics: Singlet–Triplet Conversion in Carbonyl-Substituted Anthracenes

**DOI:** 10.1021/acs.jpcb.5c07987

**Published:** 2026-02-03

**Authors:** Cesar A. Guarin, Alejandro Larios-Sandoval, Michelle Avila-Serna, Melissa Bravo-Romero, Jesús Jara-Cortés, Antonio Resendiz-Pérez, Jorge Peon

**Affiliations:** † Universidad Nacional Autónoma de México, Instituto de Química, Ciudad Universitaria, Circuito Exterior, Ciudad de México 04510, Mexico; ‡ Unidad Académica de Ciencias Básicas e Ingenierías, Universidad Autónoma de Nayarit, Tepic 63155, Mexico; § Universidad Autónoma Metropolitana, San Rafael Atlixco, Col-Vicentina, Ciudad de México 09310, Mexico

## Abstract

A new study is presented to elucidate the photodynamics of model carbonyl-substituted polyaromatics targeting the relevance of carbonyl-group orientation and torsional re-equilibration on intersystem crossing (ISC). Our experiments focused on 9-acetylanthracene (9AA) using femtosecond resolved spectroscopy. In the ground state of this molecule, steric interactions force the carbonyl substituent into a near-perpendicular orientation relative to the aromatic system. The time-resolved signals from 9AA show that ISC takes place after spectral shifts that reflect the evolution of the carbonyl group to a slanted geometry as it adjusts to a dihedral angle of around 40° with respect to the aromatic plane. Depending on the solvent, in 9AA manifold crossing takes place on the 3 to 25 ps time-scale. On the other hand, for 2-acetylanthracene (2AA) which is coplanar in both S_0_ and S_1_, the emission lifetimes can reach several nanoseconds. Analysis of these systems at the highest available theoretical levels reveals further insights into the excited-state dynamics. For 9AA and in contrast with previous publications, it is established that for all relevant geometries, the first excited singlet retains a ππ* character and decays through ISC with no involvement of other singlet states. The manifold crossing involves the interaction with the triplet manifold through states which’s transition orbitals are partially localized at the acetyl substituent. Specifically, the slanted geometry of the carbonyl group in 9AA and the potential energy surface around the equilibrium S_1_ geometry implies significant spin–orbit interactions and accelerated manifold-crossings. The present results highlight the relevance of substituent reorientation and their slanted geometries which appear to be a dominant feature in carbonyl and nitrated aromatic systems which show rapid ISC dynamics. In the article, we include details on the differences in the mechanisms operating in these two kinds of systems which show the fastest ISC rates among organic chromophores.

## Graphical abstract

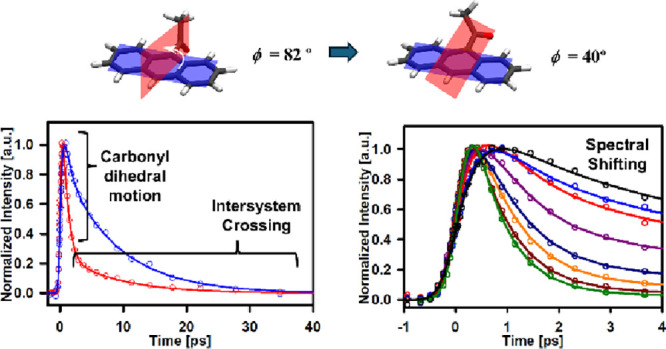

## 1 Introduction

Intersystem crossing (ISC) is a fundamental nonradiative channel in the evolution of excited states. In some cases, this channel is undesired since it limits the fluorescence yields.^1^ On the other hand, efficient crossing to the triplet states can be a target-property to form long-lived states with applications in the fields of photocatalysis and photopharmacology.^2–4^ In addition, these processes play a crucial role in systems which display delayed fluorescence where the S_1_ state can be thermally repopulated from the first triplet state which acts as a reservoir for electronically excited molecules.^5^ Finally, in the field of functional materials, ISC is also of central importance since excitonic migration and other processes can depend on the nature of the electron–hole pair.^6^

The topic of ISC is also an intense area of research from a theoretical perspective since this mechanism is intrinsically complex and can include spin-vibronic interactions as well as multielectron interactions beyond the central field approximation.^7^ Also, the ordering of the electronically excited states in molecules with an extended conjugation can depend on the type of calculation which in turn can bias the interpretation of experimental data with regard to the involvement of specific states in the ISC process. In particular, when TDDFT methods are used without significant benchmarking, the predicted state orderings and relative energies can be affected by basis-set selection and the overall treatment of the correlation and exchange interactions.^8–12^

In recent years, several investigations have shown that ISC can occur in much faster time-scales than previously considered for certain organic molecules without the involvement of heavy-atom effects. The rates for the respective decay of the initial singlet state in such cases can range from subpicosecond to a few picoseconds. Typical systems with these rapid processes include carbonyl aromatic molecules like benzophenones, xanthones, some anthraquinones; nitroaromatic compounds, and even some DNA bases.^13–19^

Of relevance for the present contribution, it has been established that for several nitrated polyaromatics, the NO_2_ atom triad lies in a plane which is tilted or oblique from the aromatic plane due to steric hindrance.^20–22^ In some of these compounds the subpicosecond to picoseconds ISC is due to spin–orbit interactions between the ππ* first singlet excited state and the second excited triplet T_2_ which can be termed as tilted-ππ* or nπ* states (see below).^23–26^ Such effect is related to the energy-coincidence of the singlet–triplet pair and to the switch-on of spin–orbit couplings related the nonparallel orientation of bonds localized in the aromatic plane and those associated with the tilted substituent double bonds.^27,28^ Similar effects related to twisted geometries have been observed in other systems including twisted acenes,^12^ core-twisted perylenediimides,^29^ and helicenes;^30^ although in these systems the rates for ISC are much smaller in comparison with carbonyl-substituted and nitrated aromatics.

In the present contribution we explore which kind of mechanism is present for other substituted polyaromatic molecules with similar noncoplanar geometries to nitroaromatics. The study of ISC dynamics of tilted carbonyl aromatic systems is particularly interesting from an electronic structure point of view. As mentioned, several instances have shown that these molecules do show rapid and efficient ISC. On the other hand, the electronic structure and dynamics of these and other systems should be intrinsically different to nitroaromatics, the other kind of substituted organic chromophores with ultrafast ISC.^20,23,26^ This difference can be described in simple terms on the basis of the local π molecular orbitals. In compounds like 9-nitroanthracene, the (T_2_) upper triplet which strongly interacts with the first excited singlet shows in calculations as a ^3^ππ* state where the respective π orbital is nitro-localized and is sometimes termed n_Oπ_ due to its nodal plane at the nitrogen atom.^31,32^ This orbital corresponds to the HOMO–1 orbital in the calculations of ref 23. On the other hand, carbonyl groups can be associated only with a C=O π-bonding and a π-antibonding orbital. This contrasts with nitrated systems, where the nitro group is associated with a π bonding orbital, the aforementioned occupied n_Oπ_ orbital and an unoccupied π antibonding orbital; that is, the 4 electrons 3 orbitals system of the nitro group.^31,32^ This difference makes it interesting to study the fast ISC dynamics in twisted aromatic carbonyls to highlight the differences in the mechanisms for efficient ISC in these two kinds of systems.

Specifically, we have studied the carbonyl-polyaromatics 9-acetylantracene (9AA) and 2-acetylanthracene (2AA). The structures of these molecules are included in Scheme 1. One of our main objectives was to study the dynamics within the singlet manifold regarding whether S_n_ → S_m_ internal conversion takes place before ISC upon excitation of the first electronic transition. Also, we seek to establish the actual time scales for their ISC step and determine the characteristics of the singlet and triplet states involved, drawing attention to the effect of the carbonyl orientation. For this, the time-resolution of the spontaneous emission from the S_1_ state reveals the rates for the decay of this state in different media. In this framework, the present studies complement and precise the interpretation of transient absorption studies with respect to the character of the involved singlet excited state and the actual rate of ISC.^33^

**Scheme 1:**
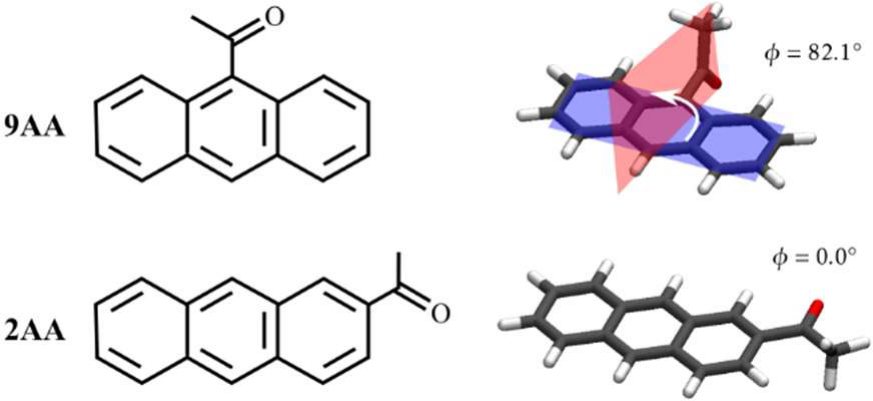
Molecular Structures of the Carbonyl-Anthracenes of This Study: 9-Acetylanthracene (9AA) and 2-Acetylanthracene (2AA)^a^

a The dihedral angle between the C–C=O plane and the aromatic plane from gas-phase XMS-RASPT2 calculations are indicated (*ϕ*).

Previous time-resolved emission studies have focused on the subpicosecond dynamics in 9AA which are dominated by rapid spectral shifting due to a reorientation of the torsional angle of the carbonyl substituent.^34^ Such breakthrough studies established the nature of early evolution within the S_1_ state but did not focus on the subsequent ISC time-scales nor the nature of the states responsible for manifold crossing in 9AA. As we show, the present results are consistent with the observations and interpretation of the subpicosecond stage but expand to the subsequent ISC dynamics. Comparisons with the 2AA molecule, which’s carbonyl group is coplanar with the aromatic system, served to illustrate the effect of the carbonyl group position and orientation on these processes.

Our study includes the use of different levels of theory to establish the state ordering of the singlet manifold at different geometries and the relevant spin–orbit couplings. Here, an exploration of how different methods treat the singlet and triplet manifolds was important to obtain results that are consistent with the static and dynamic spectroscopic signals. The results indicate that depending on the functional form employed in different TDDFT calculations, there can be systematic shifts in relative state energies and orderings in both the singlet and triplet manifolds that can bias the interpretation of transient signals which do not differentiate between emissive and nonemissive states.

Put together, the present measurements combined with previous transient absorption experiments, and a systematic computational benchmarking study using as reference the extended multistate restricted active space second-order perturbation theory (XMS-RASPT2) level give a clearer view of the nature of the ISC in this kind of molecules.

## 2 Experimental and Methods Section

### 2.1 Materials

All compounds and solvents were purchased from Sigma-Aldrich. 2-acetylanthracene (2AA) was used after repeated recrystallization from ethanol. 9-acetylanthracene (9AA) was purified by silica gel column chromatography, followed by recrystallization until samples of high purity were obtained. HPLC quality solvents were used for the spectroscopic studies (acetonitrile, cyclohexane, chloroform and methanol).

### 2.2 Steady-State Spectroscopy

Absorption and fluorescence spectra were recorded with a Cary-50 (Agilent) and a Cary Eclipse (Agilent) spectrophotometers, respectively. All steady-state experiments were performed at room temperature (20 ± 1 °C) under aerated conditions in 1 cm quartz cell. Fluorescence quantum yields were determined by the relative method using anthracene as standard in cyclohexane (Φ_f_ = 0.36 ^35^) solutions and are in excellent agreement with previous measurements.

### 2.3 Time-Resolved Spectroscopy

The femtosecond fluorescence up-conversion setup has been described previously.^36,37^ It is based on a regeneratively amplified, 1 kHz Ti:sapphire laser centered at 800 nm producing a 0.7 W pulse train of 100 fs in duration. For sample excitation, the second harmonic was used. The samples were studied in a 1 mm flow-cell, and the fluorescence was collected with a pair of parabolic mirrors and refocused to an up-conversion β-BBO crystal where it was crossed with approximately 1 mW of the fundamental beam. The sum-frequency signal was collected with a CaF_2_ lens and focused into a double 10 cm monochromator (Oriel) and detected with a photomultiplier tube. The excitation beam (400 nm) was modulated at 1/3 of the laser repetition rate with a mechanical phase-locked chopper, so that the up-conversion signal could be detected with a lock-in amplifier (Standford Research Systems). The polarization of the excitation pulses was adjusted at magic angle conditions (54.7°) with respect to the ordinary axis of the up-conversion crystal (type I sum-frequency process, detecting the vertical component of the fluorescence intensity). The instrument response function (IRF) for the up-conversion experiments was determined to be Gaussian with a full width at half-maximum of 450 fs through the resolution of solvent-Raman signals. Decays at different temperatures from 276.15 to 323.15 K were performed using a cooling station where the flowing solution was placed in contact with a refrigeration unit, measuring the temperature at the solution reservoir. Time-resolved anisotropy measurements (*r*(*t*)) were made through the traces obtained with a parallel and perpendicular orientation of the excitation beam with respect to the vertical (ordinary) angle of the up-conversion crystal.

For the time correlated single photon counting (TCSPC) technique we used a confocal setup, with a 354 nm picosecond laser for excitation (LDH-P-FA-355, 354 nm, 48 ps fwhm, PicoQuant).^38^ The fluorescence was collected, collimated and focused to a 50 μm avalanche photodiode (PD-050-CTE, Micro Photon Devices), which was connected to a TCSPC-system (Pico Harp 300, PicoQuant), and synchronized with the laser repetition rate. A dichroic mirror (Chroma 425dcxr) was used to eliminate residual excitation photons. No signal was detected with blank solutions. The IRF was determined with a fluorescein solution at pH 10 saturated with KI. Fluorescence lifetimes were determined with the SymphoTime 64 software (PicoQuant) using the Levenberg–Marquardt iteration algorithm.

### 2.4 Computational Methods

Extended multistate restricted active space second-order perturbation theory (XMS-RASPT2) and TDDFT gas-phase calculations were performed since these are the highest available computational methods for this molecular size. A systematic benchmarking study was made in order to test for the most appropriate TDDFT method for solution systems for the singlet and triplet manifolds considering the gas phase XMS-RASPT2 results and the experimental data. The XMS-RASPT2 geometry optimizations using analytical gradients were performed with the MA-def2-SVP basis set.^39^ The restricted active spaces comprised 6 (RAS-1), 6 (RAS-2) and 5 (RAS-3) orbitals, and a total of 18 electrons. For the benchmarking studies, the following functionals were tested against the gas phase results from XMS-RASPT2: BLYP, PBE, TPSS, TPSSh, B3LYP, PBE0, TPSS0, BHHLYP, ωB97X, and CAM-B3LYP. Also, the use of different basis sets was tested (see results section). For all functionals we tested the trends for the triplet and singlet energies considering a Full Linear Response scheme (FLR) and the Tamm-Dancoff approximation (TDA). Such benchmarking was made given that it has been observed that FLR methods can show significant artificial shifts of the triplet state to lower energies related to the well known triplet-instability issue of FLR methods.^8,10,40^ This is particularly important for dynamical systems where ISC is being studied due to the central role played by the couplings of the first excited singlet and specific higher triplet states, and the specific energetic ordering of the states within each manifold (in particular, the singlet manifold, see below). Comparisons between the XMS-RASPT2 results and those of different TDDFT schemes are included in the Supporting Info. and discussed in the results section. The comparisons with the available experimental spectra were made to determine the accuracy of the selected TDDFT method in different solvents.

For comparisons with experimental data, the absorption and emission spectra simulated with TDDFT were obtained using two approaches. The first involved convoluting the stick spectra with Gaussian functions, followed by averaging across different structures obtained from configurational sampling around the S_0_ or S_1_ equilibrium geometries using the Wigner distribution at 298 K.^18,41,42^ The second method employed the Fermi golden rule framework in which the vibrational contribution to the wave functions was treated within the harmonic approximation, based on frequencies and normal modes (*Q_i_*) associated with the S_0_ and S_1_ minima. Electronic and vibrational interaction was accounted for by expanding the transition dipole moment in a first-order Taylor series with respect to *Q_i_* (Herzberg–Teller couplings).^43^ These predicted vibroelectronic spectra can be used to assess the precision of these calculation by direct comparisons with experimental spectra which show clear vibronic progressions, as is the case for 9AA and 2AA (including their emission spectra in cyclohexane, see below). Spin–Orbit Coupling (SOC) values between relevant states were estimated from a one-electron Breit–Pauli operator. The multiconfigurational XMS-RASPT2 calculations were performed with the OpenMolcas electronic structure code, and the TDDFT and computations were carried out with Orca 6.0.^44,45^

## 3 Results and Discussion

### 3.1 Steady-State Spectroscopy

In Figure 1 we include steady-state absorption and emission spectra of both compounds in a series of solvents. 9AA presents vibroelectronic structured spectra in all solvents, similar to the anthracene parent compound, where the first transition at 382 nm is only 5 nm red-shifted in comparison to anthracene. This behavior is similar for 2AA which shows a more pronounced structure in cyclohexane. While the 2AA solutions show intense fluorescence signals, with quantum yields from 0.04 to 0.8, 9AA is a minimally fluorescent compound at room temperature (for example, Φ_f_ = 2 × 10^–4^ in cyclohexane, see Table 1) which is typical for molecules with a fast S_1_ depopulation channel. Due to the low quantum yields of 9AA, we include the excitation spectra for this molecule in the Supporting Info. (Figure S1). Despite the low steady-state emission signals, the presence of the same vibro-electronic progression pattern in the excitation and absorption spectra allowed the verification of the source of this weak emission, confirming that it corresponded to 9AA and not to impurities or photoproducts. Such emission spectra could then be used to scale the time-resolved emission signals to reconstruct the spectral evolution (see below). For 9AA, we have included chloroform in the solvent set since transient absorption data for this molecule is only available from the literature in this solvent. Table 1 summarizes the steady-state results and other photophysical parameters, and Table S1 shows comparisons with previously reported values of emission yields showing excellent agreement.

**Figure 1:**
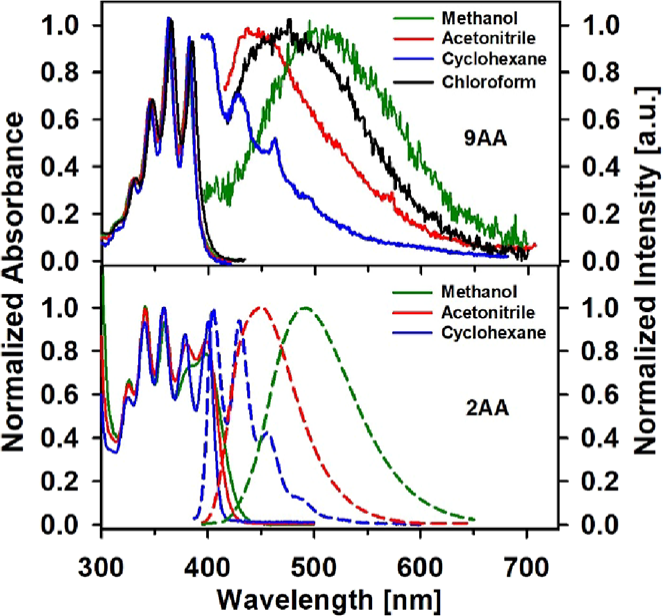
Normalized steady-state absorption and fluorescence spectra for carbonyl compounds 9-acetylanthracene (9AA) and 2-acetylanthracene (2AA) in polar and nonpolar solvents. The excitation wavelength was 365 nm for 9AA and 360 nm for 2AA.

**Table 1:** Spectroscopic and Photophysical Data for 9AA and 2AA^a^

**molecule**	**solvent**	**Φ_f_**	**λ**_**f**_ **(nm)**	**Φ**_**T**_	**S**_**1**_ **(fluorescent-state) lifetime, τ**	**k**_**ISC**_ **(s** ^**‑1**^ **)**
9AA	cyclohexane	4.4 × 10^–5^	426	0.97^b^	3.4 ps	2.85 × 10^11^
	acetonitrile	2.0 × 10^–4^	445	1.00^b^	8.0 ps	1.25 × 10^11^
	chloroform	<0.01^b^	475		23.8 ps	
	methanol	1.8 × 10^–3^	503	0.69 ^a^	25.7 ps	2.68 × 10^10^
2AA	cyclohexane	0.036	430		1.04 ns	≤9.6 × 10^8^
	acetonitrile	0.53	448		11.32 ns	≤8.8 × 10^7^
	methanol	0.78	493		15.02 ns	≤6.7 × 10^7^

a Φ_f_: fluorescence quantum yield, λ_f_: maximum fluorescence wavelength, Φ_T_: quantum yield for triplet formation, k_ISC_: intersystem crossing rate.

b Quantum yields for triplet formation of 9AA were taken from ^a^ref 46 and ^b^ref 33. The k_ISC_ values for 2AA are indicated as upper limits since the triplet yields are not available. These upper limits correspond to the total nonradiative rates and were calculated from the total S_1_ decay rates and the radiative rates which in turn were calculated from the total decay rate and the fluorescence yields. Only relative values for triplet yields in 2AA have been reported and correspond to 0.3 and 0.01 for acetonitrile and methanol respectively with respect to that in cyclohexane.^47^

### 3.2 Time-Resolved Spectroscopy

#### 3.2.1 9-Acetylanthracene (9AA)

The time-resolved fluorescence traces for 9AA are presented in Figures 2A (cyclohexane), 2B (chloroform), S2 (acetonitrile), and S3 (methanol). In Figure 3, we include the evolution of the emission spectra reconstructed from single-wavelength traces.^48^ For all solvents, the signals evolve in different time-scales: a subpicosecond component is present and implies a rapid reduction of the emission intensities on the blue side of the spectra and rises on the red side. The time constants for the fast components range from 0.3 ps in acetonitrile to 0.6 ps in chloroform. Except for cyclohexane, an intermediate component of a few ps is also present, representing a slower component for the red shifting of the spectra which has been assigned to vibrational energy redistribution, and solvent response within the emissive state.^34^ After this evolution, the emission across the spectra for all systems decays to the baseline with time constants of 3.4 ± 0.6 ps in cyclohexane, 8 ± 1 ps in acetonitrile, 23.8 ± 2.3 ps in chloroform and 25.7 ± 3 ps in methanol. Several of the traces were taken both with 380 and 400 nm excitation, and presented the same time constants and amplitudes within experimental error. The signal description was made through joint fitting of the data across the spectrum considering globally fitted values for the different time constants. This procedure produced adequate signal descriptions across the spectra for all systems as can be seen in Figure 2. The different amplitudes and time constants are included in the Supporting Info. in Tables S2–S5, while Table 2 compiles the time constants for 9AA (and 2AA) in each solvent.

**Table 2:** Lifetime of the S_1_ Excited State for 9AA and 2AA

**molecule**	**solvent**	**τ**_**1**_ **(ps)**	**τ**_**2**_ **(ps)**	**τ**_**3**_ **(ps)**
9AA	cyclohexane	0.4		3.4
	acetonitrile	0.3	0.9	8.2
	chloroform	0.6	2.3	23.8
	methanol	0.5	2.8	25.7

				**τ**_**3**_ **(ns)**
2AA	cyclohexane	0.3	19.1	1.04
	acetonitrile	0.5	21.8	11.32
	methanol	4.9	16.7	15.02

**Figure 2:**
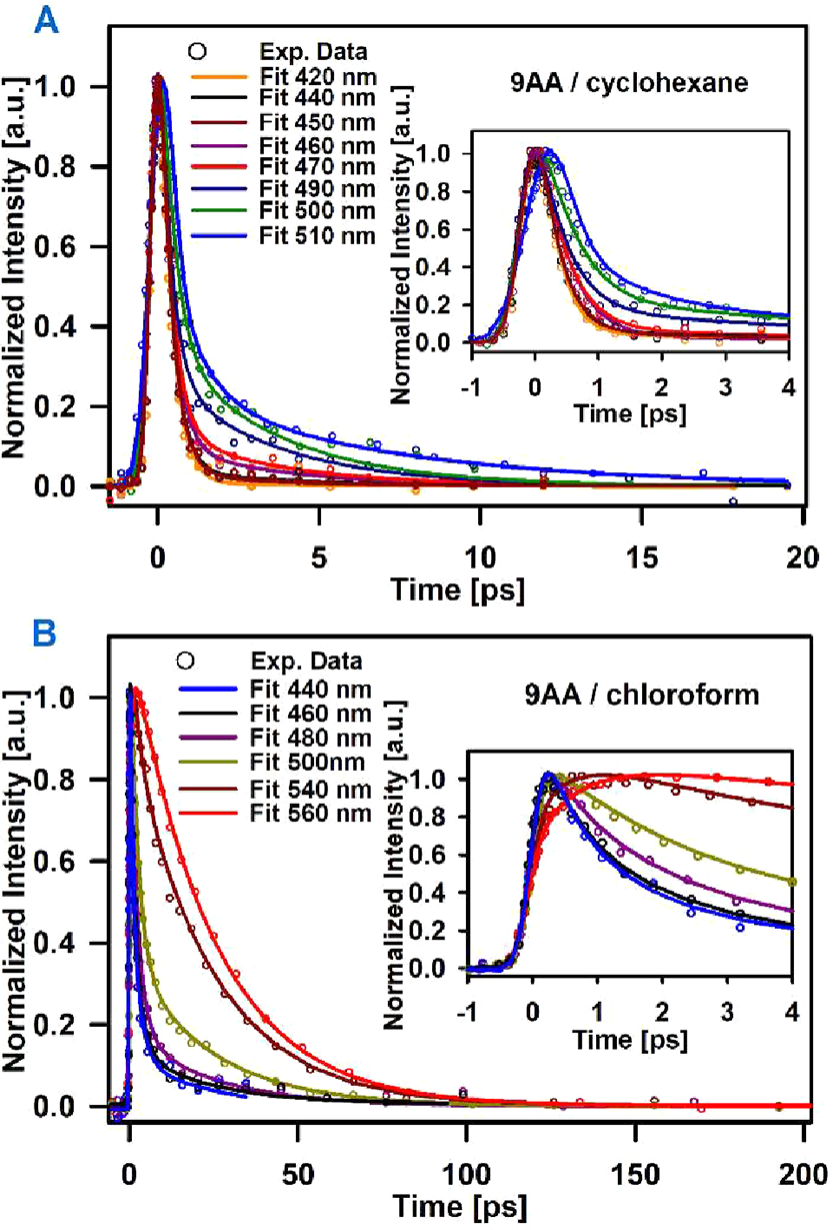
Single-wavelength time-resolved fluorescence for 9AA in different solvents. (A) Cyclohexane, (B) chloroform. The inset shows the early evolution of the same traces.

**Figure 3:**
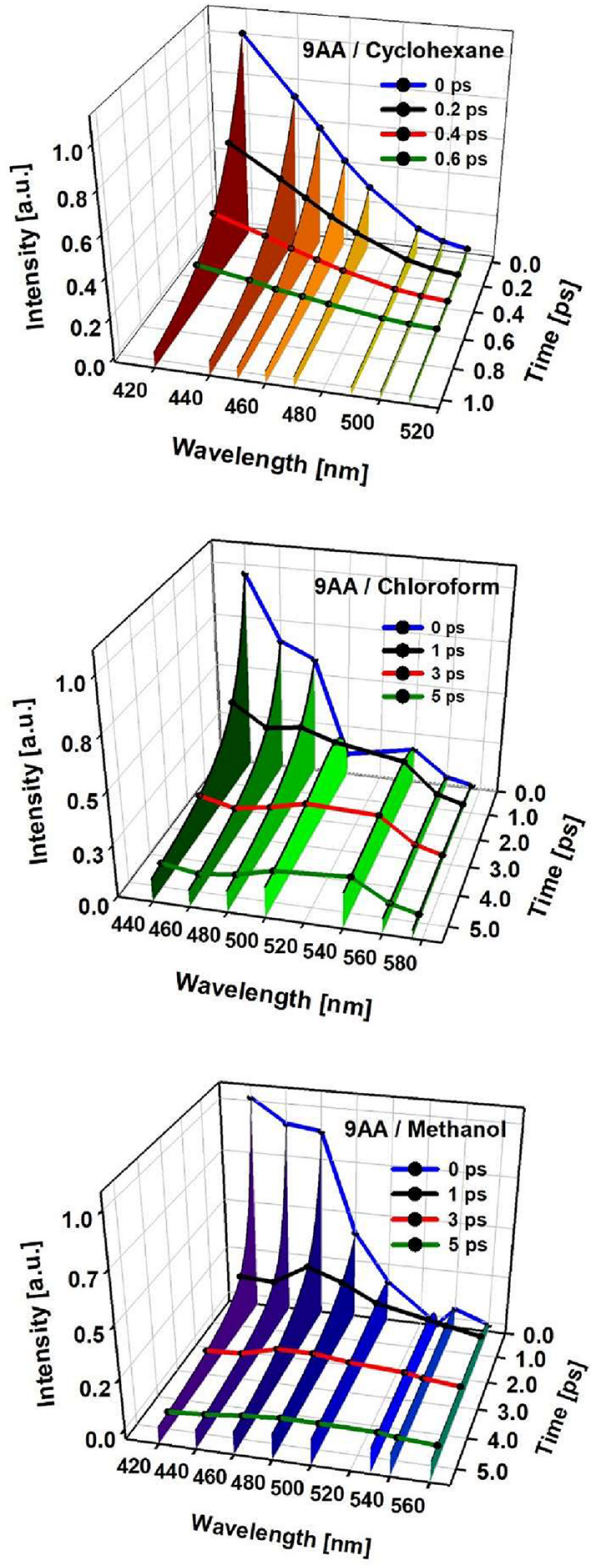
Time-resolved emission spectra reconstructed from the femtosecond up-conversion transient fits for 9AA in cyclohexane (top), chloroform (middle), and methanol (bottom).

The ultrafast dynamics of 9AA have been studied previously in the seminal work by Barbara and co-workers^34^ For this time scale (faster components), it can be established that the rapid signal decays on the blue side of the spectra corresponds to a red shifting of the emission spectrum which can be appreciated in greater detail than previously in Figure 3. These dynamics have been associated with the evolution of the locally excited (LE) S_1_ state toward a different carbonyl-dihedral geometry with a larger charge transfer character.^34^ In fact, the ground state of 9AA shows a near perpendicular orientation of the acetyl group with respect to the polyaromatic plane (87.9° in crystalline phase,^49^ while our calculations indicate that the equilibrium geometry in the S_1_ state corresponds with a dihedral angle of nearly 40° (see below)). As can be seen, the time-resolved spectral profiles of Figure 3 show that the shift of the emission maximum takes place before the subsequent emission decay to the baseline. The time scales for the torsional evolution process have been shown not to correlate with the solvent viscosity indicating that the torsional motion time-scale is primarily defined by intramolecular friction terms related to intramolecular vibrational redistribution (IVR).^34^ The traces of the present study are consistent with these previous ultrafast results and explanations about the early dynamics but show the full spectral evolution.

After the early evolution, the decay of the emissive S_1_ state is dominated by ISC. This can be concluded from the observation of the T_1_ absorption spectrum from a few tens of picoseconds up to the nanosecond regime in transient absorption experiments.^33^ Additionally, large yields for triplet formation have been reported for 9AA in cyclohexane, acetonitrile and methanol (0.97 ± 0.04, 1.00 ± 0.04 and 0.69 ± 0.04 respectively).^46^ As can be seen, the time-scale for the S_1_ decays ranges from 3.4 ps (cyclohexane) to 25.7 ps (methanol). A likely relevant issue with respect to the S_1_ decay times is that they are in some cases clearly shorter than vibrational cooling times observed for several solute/solvent systems (up to 35 ps in cyclohexane,^50^ and 13.1 ps for acetonitrile^51–53^ ). While vibrational cooling depends on the specific solute, comparisons of these time-scales with the S_1_ lifetimes suggest that full vibrational cooling may not be completed in the observed time-scale of ISC. In order to test whether the ISC step is activated and temperature-dependent, we performed fluorescence up-conversion measurements from 276.15 to 323.15 K in the four solvents. We observed only for the case of chloroform (Figure S4) a slight ISC temperature dependence (from 27.9 ps at 276.15 K to 22.4 ps at 323.15 K), while for the other solvents, the decays only vary within our time-constant uncertainty (±1 to ± 3 ps). Such minimal or absent temperature dependence indicates that ISC in these systems may include still vibrationally hot molecules in the S_1_ state as has been observed for other surface crossing phenomena.^54–56^

As mentioned, the photodynamics of 9AA have also been studied by transient absorption.^33^ Such study was performed only in CHCl_3_ and observed a spectral evolution which is accurate and of excellent quality. However, the dynamical interpretation from the transient absorption study should be reconsidered in view of the current results which show that the emission signals (after the initial torsional evolution) decay with a time constant of 23.8 ps in this solvent. The original analysis of the transient absorption experiments was made with the aid of singular value decomposition and showed the presence of a 290 fs component, which was followed by a 10 ps component giving rise to the long-lived absorption spectrum of the T_1_ phosphorescent state which is well-known from previous studies.^57^ The original interpretation given to these processes considered that the ultrafast component corresponded to ISC while the 10 ps component was related to relaxation within the triplet manifold. The present time-resolved emission experiments for 9AA in CHCl_3_ show that the originally excited state evolves through the carbonyl dihedral coordinate, and that the emission signals then decay with a time constant of 23.8 ps in this solvent. This indicates that ISC is the dominant process present in the *t* ≥ 10 ps regime, and that the subpicosecond components are related to carbonyl reorientation within the S_1_ state. Our assignment of 23.8 ps for crossing to the triplet manifold is also consistent with a previous measurement which associated this process with the 20 ± 4 ps rise of transient absorption signals from the T_1_ state.^58^ As we show in the computational section, the evolution of the emission spectra of Figures 2, 3, S2, S3 and S5 are consistent with such assignment and are also consistent with the transient absorption data from the previous study despite a relevant revision of the assigned dynamics.

The present time-resolved studies also clarify that the emissive state originally populated by optical excitation remains the lowest energy (fluorescent) singlet excited through the initial relaxation dynamics and during the ISC step. In particular, the previous TDDFT calculations with the B3LYP functional (6–31G­(d,p) basis set^33^) predicted that after excitation rapid internal conversion would populate a nπ* singlet which would become the lowest energy excited singlet as the molecule evolved toward equilibrium within the singlet manifold.

In order to further confirm the lack of any singlet–singlet state crossings during the relaxation revealed by the spectral evolution, we performed time-resolved anisotropy studies for 9AA in cyclohexane and chloroform. The results are included in Figure 4. As can be seen, the fluorescence anisotropy (*r*(*t*)) traces do not show any rapid changes as the system evolves toward the equilibrated spectrum indicating that the emission transition dipole orientation does not change during the spectral evolution (noting that the S_1_ and S_2_ states in this system have perpendicular transition dipole moments, see computational section). The slower decay in *r*(*t*) can be readily assigned to orientational diffusion (approximately 30 ps). Such results further illustrate that the originally excited emissive singlet remains populated and that no internal conversion between excited singlets is observed before or during the emissive state decay from ISC. The lack of state-crossings within the singlet manifold before ISC is also well supported by the present computational results which include comprehensive benchmarking for several TDDFT functionals and methods (computational section). In summary, the up-conversion data of 9AA indicate that the actual time-scale for the lifetime of the S_1_ state after carbonyl reorientation ranges from 3.4 to 25.7 ps, and specifically, for chloroform is 23.8 ps. Table 1 includes these lifetimes and respective values for the ISC rate constants for the cases where the triplet quantum yields are known.^46^ These rates are of the order of 10^11^ s^–1^ and were calculated as the ratio of the triplet yields and the S_1_ lifetimes.

**Figure 4:**
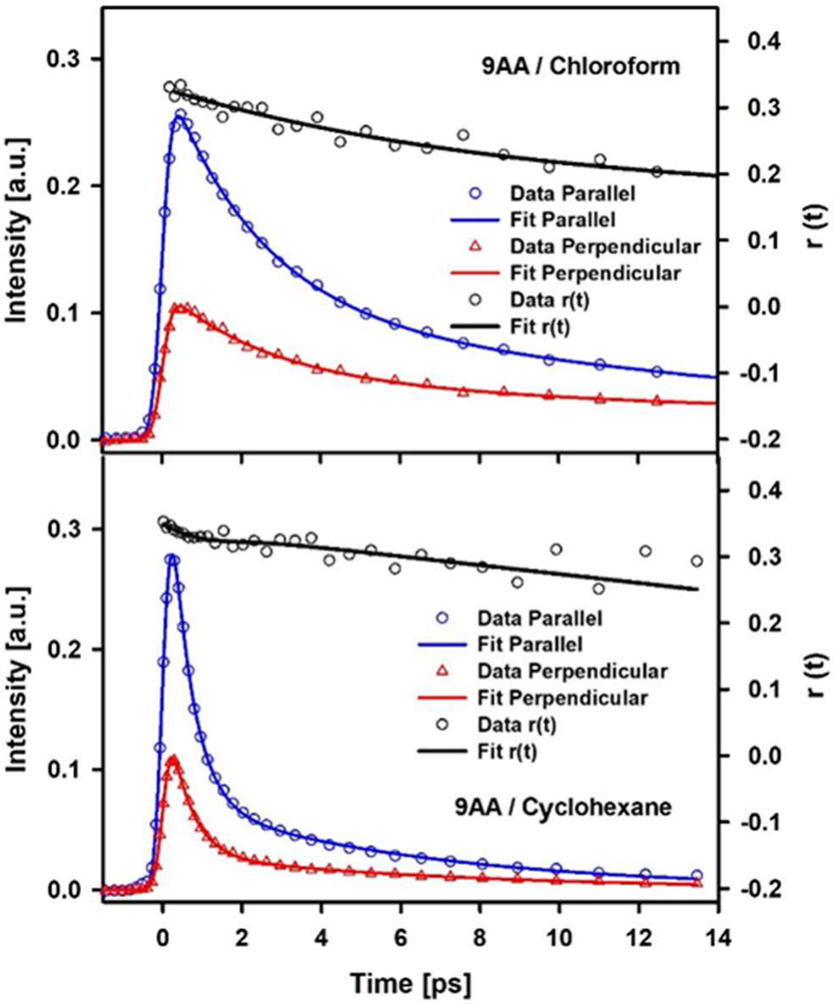
Time-resolved fluorescence anisotropy measurements for 9AA cyclohexane and chloroform. The parallel data corresponds to traces obtained when the excitation polarization was parallel to the detection axis, and the perpendicular data were obtained with perpendicular orientation of the excitation beam. The black line corresponds to anisotropy values calculated from the convoluted fits to the parallel and perpendicular traces.

#### 3.2.2 2-Acetylanthracene (2AA)

2AA shows completely different dynamics in comparison with 9AA which can be directly related to the difference in the orientation of the acetyl group with respect to the polyaromatic plane and the position of this substituent. As we show in Figures 5, 6 and S6 in all solvents, the emissive state is much longer-lived, reaching several nanoseconds for the S_1_ lifetime (summary in Table 2). Such slow decay is better seen from TCSPC measurements as included in Figure S6. Depending on the solvent, the emission lifetime is preceded by modulation of the spectral maximum position which can be assigned to solvation and vibrational relaxation. This much slower S_1_ decay for 2AA is fully consistent with the previous transient absorption measurements.^33^ In particular, for the case of methanol, the typical solvation dynamics are manifested by the amplitude rise of the signal on the red side of the spectrum, a typical signature of the diffusional solvation time component in this solvent (15 ps^59^). The drastic difference in the S_1_ decay in comparison with 9AA can be associated with a much smaller coupling with the triplet manifold which is in turn related to the coplanarity of this substituent with the aromatic system in both the S_0_ and S_1_ states.^33^ This is further described in the computational section.

**Figure 5:**
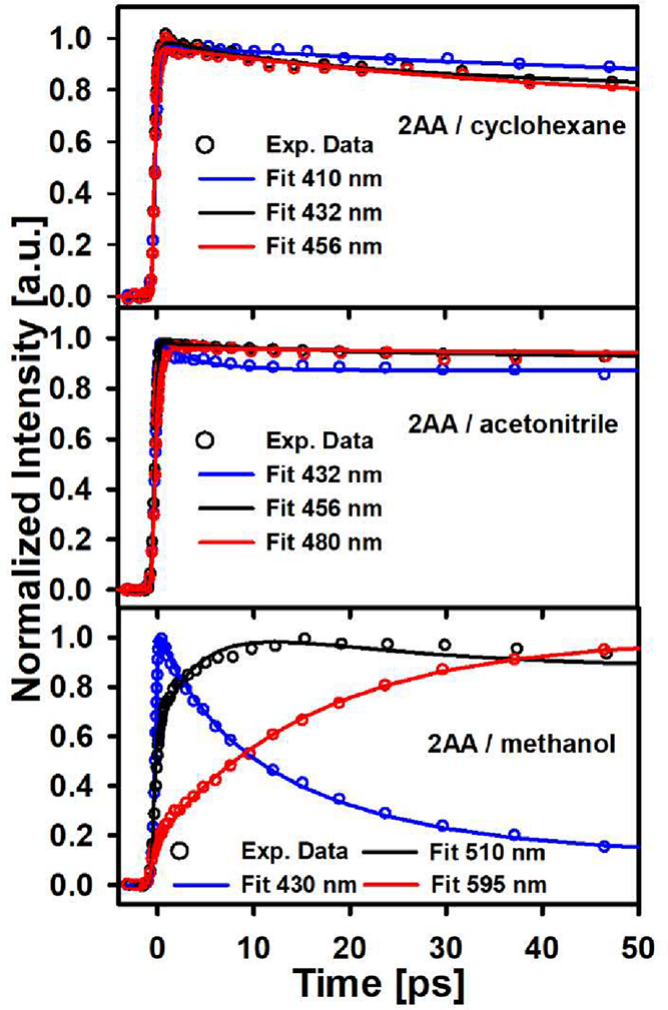
Single wavelength time-resolved fluorescence for 2AA in different solvents.

**Figure 6:**
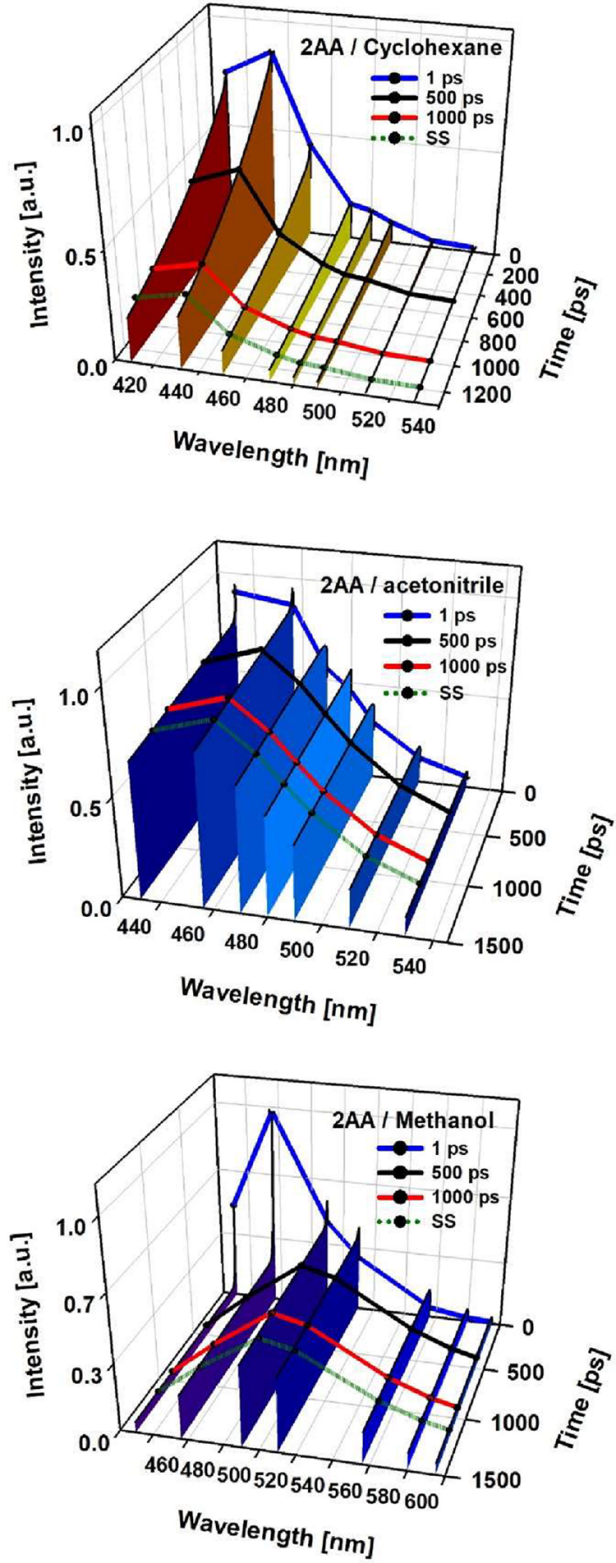
Time-resolved emission spectra reconstructed from the femtosecond up-conversion transient fits for 2AA in cyclohexane (top), acetonitrile (middle), and methanol (bottom). The green line shows points in the steady-state (SS) spectrum.

### 3.3 Computational Results

#### 3.3.1 XMS-RASPT2 Results and Benchmarking for TDDFT Methods

As mentioned in the methods section, a benchmarking study was made to search for the best TDDFT method and basis set to apply with regards to the relative energies and characters of the calculated singlet and triplet manifolds in solution. This benchmarking used as reference the results from gas-phase XMS-RASPT2 calculations at the ground state and S_1_ equilibrium geometries and include comparisons with experimental data for the solution-TDDFT calculations. The results are further detailed in the Supporting Info., and summarized in Figures S7–S15 and Tables S9–S15. This benchmarking included comparisons between FLR methods and the TDA approximation for TDDFT, searching for the most consistent methods to calculate the relative energies within the singlet manifold, and the relevance of the triplet states that could mediate ISC.

For 9AA, the reference calculation (XMS-RASPT2) in the gas phase indicated that the dominant transition configurations at the experimental photon energy correspond with a ^1^L_a_ (ππ*, transverse polarization) state as the first excited singlet (3.30 eV transition energy), while a second singlet (^1^L_b_, ππ* longitudinally polarized transition) lies 0.36 eV above. Several triplet states appear at relevant energies, including the first and second triplets of ππ* character (^3^L_a_,^3^L_b_), and higher triplets where a third triplet shows a partial ^3^nπ* character, and a fourth one with a ππ* character. The transition energies and relevant orbitals are included in Table S9 and Figure S7. The results for the 2AA system are included in Table S10 and Figure S8 and again show the ^1^L_a_ (ππ*) as the first excited singlet, with a different order with regards to the nπ* and L_b_ states in both manifolds.

When the energies of these states at the same geometry are calculated in the gas phase considering a variety of density functionals, the resulting excited state energies have significant variations from method to method, for both FLR and TDA methods. These comparisons are presented in Figures S11–S16. In particular, the FLR method clearly underestimates the energy of the triplet states as the triplet manifold appears shifted to lower energies with respect to the first excited singlet in comparison with the XMS-RASPT2 results. On the other hand, when the TDA approximation is made, the triplet state energies are in better agreement with those of the reference XMS-RASPT2 method and the relevant experimental data (phosphorescence spectra, see Supporting Info.). Of relevance for the present study, the energies of the calculated triplet states with the (TDA) CAM-B3LYP provides overall appropriate results in comparison with the XMS-RASPT2 method for the triplet manifold in the gas phase. This effect has been seen for several organic molecules of similar sizes and is related to the well-known triplet instability issue with FLR methods.^8,40^ On the other hand, the energies and ordering of the singlet excited states is better reproduced with the FLR method in comparison with the reference method. Considering these benchmarks, it was decided to compare the experimental results in solution with the results from the CAM-B3LYP functional (solvent effects were included through the PCM method) where the relative energies within each manifold were taken into account considering both FLR and the TDA methods for the systems in solution. Spin–orbit couplings were calculated with both FLR and TDA methods (both triplet and singlet manifolds using the same functional), showing the same results. Once these functionals were selected as most appropriate, the basis-set options were evaluated. We found that there is practically no effect of using increasingly expanded basis sets as shown in Figure S12, so the MA-def2-SVP set was kept for the rest of this study.

#### 3.3.2 S_1_ Surface and Intersystem Crossing Mechanism: Evolution of the S_1_ State and ISC in 9AA

The TDDFT calculations at the equilibrium geometry of the ground state (calculated carbonyl dihedral angle: 82°) show the presence of several higher triplet states (T_n_, *n* > 1) for both molecules at relevant energies. For 9AA in the gas phase, both the^3^L_b_ and a ^3^nπ* states are at similar energies of the S_1_(^1^L_a_) state. Importantly, as shown in the Supporting Info., these triplet states are energetically situated above the ^1^L_a_ singlet when the solvent effects are included, retaining this order in all solvents at the S_0_ geometry. Most relevant for the present study and fully consistent with the observed dynamics, the energy of the S_1_(^1^L_a_) state at its equilibrium geometry clearly shifts to lower values (shifting for example, by 0.38 eV in acetonitrile) and remains below the energy of all the higher triplets (T_n_, *n* > 1) and other excited singlet states. This reflects the time-resolved experiments of the present study which indicate that carbonyl-dihedral early evolution in the S_1_ state takes place before ISC (although not necessarily full vibrational cooling as mentioned). For the S_1_(^1^L_a_)-optimized geometry, the specific order of the higher states in the triplet manifold depends on the solvent, where in the gas phase the ^3^L_b_ and ^3^nπ* states are nearly degenerate, in cyclohexane the ^3^nπ* lies below the ^3^L_b_ state, while this state remains the second triplet in acetonitrile and methanol.

Figure 7 shows the relative energies of the singlet and triplet manifolds as a function the carbonyl torsion coordinate of the S_1_(^1^L_a_) state where the rest of the degrees of freedom have been optimized (relaxed scan of the carbonyl dihedral angle). The experimental results indicate that the originally excited state rapidly evolves to geometries near the equilibrium angle for this mode (40.7° gas phase, 40.4° cyclohexane, 38.38° methanol, and 38.41° acetonitrile). This geometry corresponds to the center of the horizontal axis in the curves of Figure 7. It should be noted that these adiabatic curves label the triplet states at each geometry in order of their energies at each geometry, and that the partial characters of each of these triplets depend on the geometry. In the relaxed scans of Figure 7, for all media, the equilibrium S_1_ state remains at lower energies along the torsional mode than all the higher triplets. This is fully consistent with a shifting of the S_1_ emission spectra (Figure 3) observed experimentally, and the subsequent decay of the emission signals which takes place in 3.4 to 25.7 ps depending on the solvent. As mentioned in the experimental section, the time-constants observed for the emission decay are shorter than reported measurements of vibrational cooling. This implies that although the spectral maximum shifts rapidly, it is expected that the excited molecules (S_1_ state), have not fully vibrationally relaxed as the system further evolves through ISC. This indicates that the observed ISC rates may not correspond to a thermally activated process but rather may depend on the remaining internal vibrational energy of the solute.

**Figure 7:**
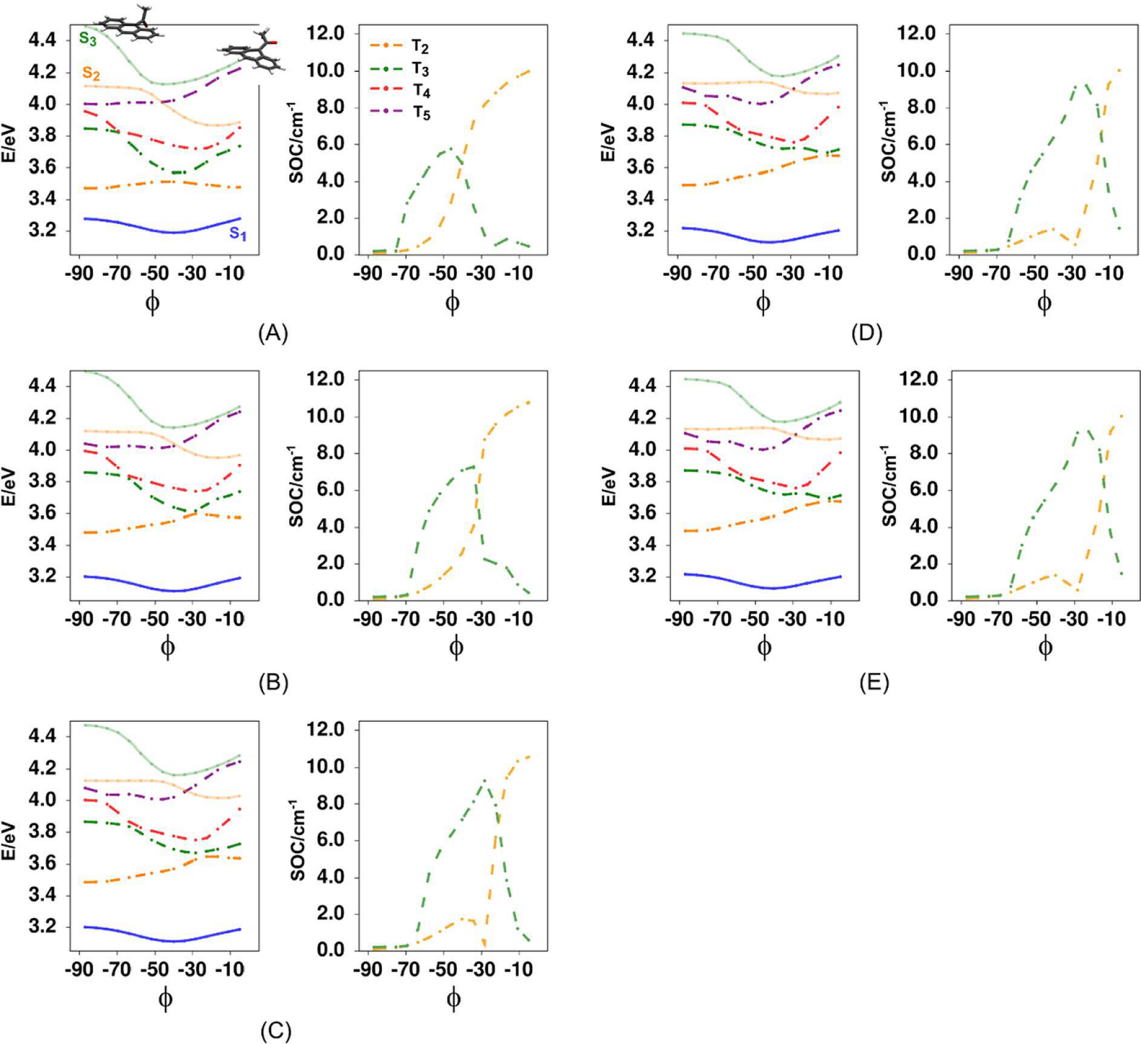
Relaxed scans (E/eV) for the ^1^L_a_ state, as a function of the OCCC dihedral angle (ϕ), as well as the corresponding spin–orbit coupling values (SOC/cm^–1^), between ^1^L_a_ and the T_2_ and T_3_ triplets in 9AA. The energy values are referenced to the minimum of S_0:_ (A) gas phase, (B) cyclohexane, (C) chloroform, (D) methanol, and (E) acetonitrile. The energies of the T_4_ and T_5_ states are included as the dashed red and purple lines. From their larger energy gaps with the S_1_ state, these triplets are not expected to participate in the ISC dynamics.

We have calculated the respective spin–orbit couplings at these geometries and included them in Figure 7, noting that these are only the coupling electronic matrix elements not involving factors related to Franck–Condon or activation coefficients which are assessed qualitatively through the inspection of the potential energy curves. As can be seen, the spin–orbit couplings increase drastically as the carbonyl group moves away from the near perpendicular orientation (S_0_ geometry) toward the equilibrium S_1_ (^1^L_a_) conformation in all media (near 40°). As this takes place, the energy gap between S_1_ and the T_2_ triplet increases and the gap to the T_3_ state decreases. Also, along the torsional coordinate near the equilibrium positions, there are clear avoided crossing angles where the T_2_ and T_3_ states switch characters. We have searched the lower frequency normal modes to explore the dependence of these motions on the energy gaps and the singlet–triplet couplings. These results are included in Figure S19 and show that for these normal modes in 9AA the energy gaps do not decrease along these coordinates. On the other hand, from explorations of the coordinates that connect the minima of the S_1_(^1^L_a_) state to the minima of the most relevant triplet states, it is observed that there are S_1_-triplet crossing points. This is shown in Figure 8 where again the adiabatic curves are labeled according to their energy order. As can be seen for the case of the triplet with larger^3^L_b_ character (T_2_ at its equilibrium geometry) the spin–orbit coupling with this state remains below 1 cm^–1^. On the other hand, for the adiabatic trajectory that crosses the triplet with larger carbonyl-nπ* character, the coupling which is already high at the S_1_ equilibrium geometry shows a further increase along the path toward the crossing point. It should be noted that along this trajectory there is an avoided crossing between the two lower lying (*n* > 1) triplets (at 0.6–2 minimal energy path units, where the T_2_ triplet switches character), so the T_2_ state has significant nπ* character near the singlet–triplet crossing. This characterization of the potential energy surfaces indicates that isoenergetic and highly coupled conditions can take place directly with the originally populated S_1_(^1^L_a_) of ππ* character without the involvement of other singlet states. The S_1_–T_2_ crossing point in this trajectory lies above the S_1_ equilibrium energy by 0.07 eV in the gas phase, 0.2 eV in cyclohexane, and 0.27 eV for the cases of acetonitrile, chloroform and methanol which is consistent with the fastest ISC observed for 9AA/cyclohexane. Taking into account typical TDDFT errors (of the order of 0.1 eV) and considering that the systems may not have fully undergone vibrational cooling, the coupling of the S_1_ state with this T_2_ triplet is the most immediate explanation for the rapid (3.4–25 ps) ISC observed for 9AA.

**Figure 8:**
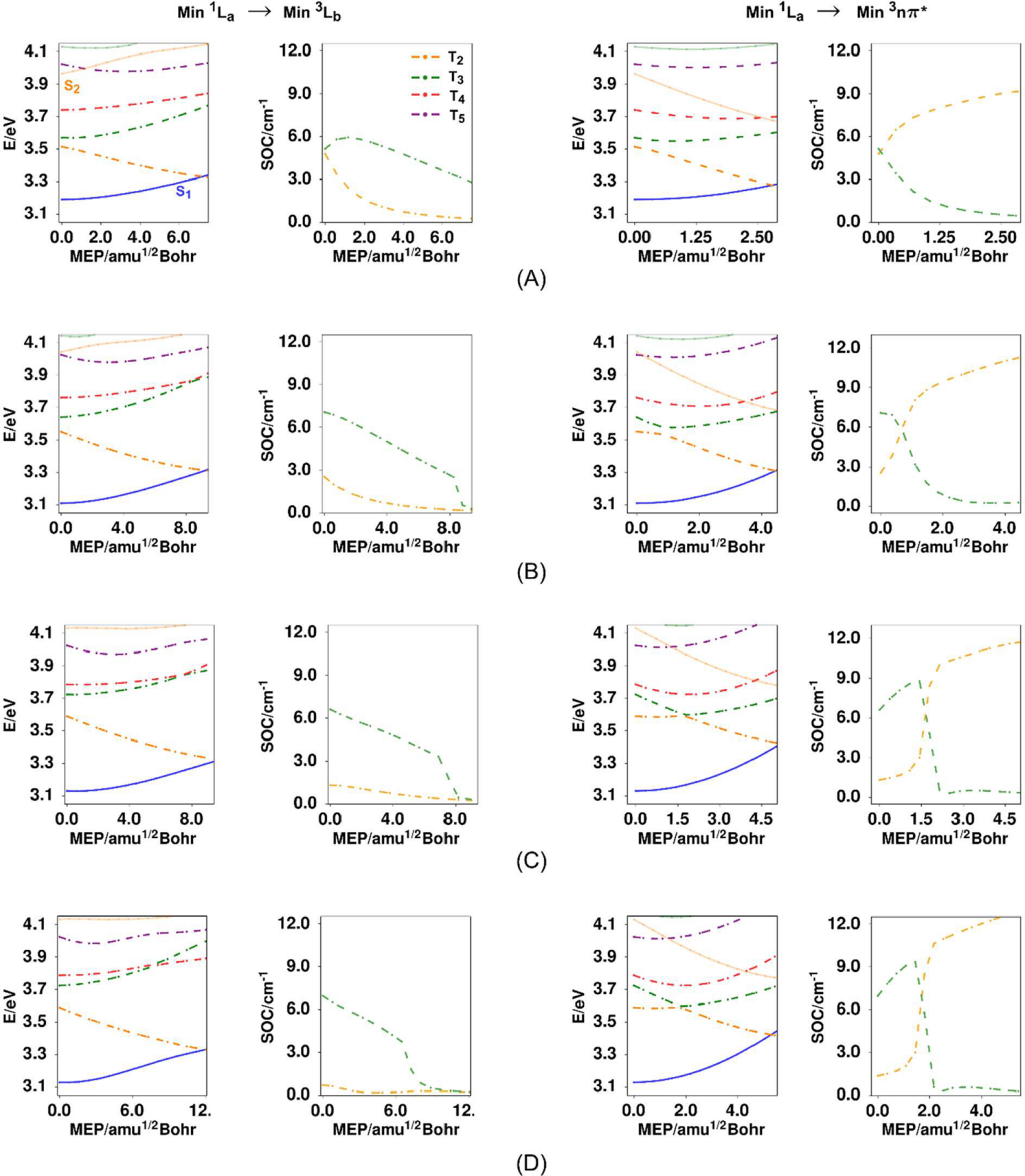
Energies (*E*/eV) along the minimum energy path (MEP) of low-lying electronic states for 9AA, as well as the corresponding spin–orbit couplings (SOC/cm^–1^), along the coordinate connecting the minimum of ^1^L_a_ with the minima of the ^3^L_b_ (graph pairs on the left) or ^3^nπ* states (graph pairs on the right) for (A) gas phase, (B) cyclohexane, (C) methanol, and (D) acetonitrile. The structures were obtained by linear interpolation in internal coordinates. The energy values are referenced to the minimum of S_0_.

Most importantly, the analysis of 9AA’s T_2_ state indicates that near the S_1_(^1^L_a_) crossing angles, this triplet involves transitions from orbitals that are partially localized at the carbonyl group (mixed with anthracenic delocalized π orbitals see orbitals H-3 to H-1 in chloroform in the Supporting Info.). As will be seen from comparisons with the 2AA case, the oblique angle of the carbonyl group drives this kind of effect which redounds in the relative lowering of the T_2_ triplet and the significant spin–orbit couplings. From an electronic and geometric structure viewpoint, this feature appears to be a principal factor for the rapid ISC in 9AA. Additional dynamic effects for manifold crossing are also likely directly related to the rapid ISC in this molecule. These can include vibrational spin–orbit and triplet vibronic mixing type of mechanisms for intersystem crossing.^7^ The respective terms can be suspected to add up to the overall crossing rates since the spin–orbit couplings show rapid increases (in particular along the torsional coordinate) together with small gaps between T_2_ and T_3_. This subject is not further explored in this contribution given its experimental and general computational description of the system.

#### 3.3.3 Case of 2AA, Reduced Couplings with the Higher Triplets

The same scanning across the substituent orientation was performed for 2AA and the results are shown in Figure 9. This system contrasts with that of 9AA in several regards. First, due to the much smaller steric hindrance of the acetyl substituent, the equilibrium geometry for both the ground state and the ^1^L_a_ emissive state correspond with a coplanar arrangement between the carbonyl group and the aromatic plane, also having a more curved (steeper) form in comparison with 9AA. Again, no crossings were detected along this scan with any of the higher triplets along the torsional coordinate nor in the respective modes explored for 9AA. In this case, only two triplets show nonzero SOC couplings. The T_2_ and T_3_ triplets do not show crossings along this coordinate, and for both states, the transition orbitals are almost fully localized at the aromatic rings, defining a ππ* character for these triplets. In fact, at the equilibrium geometries the couplings are zero for both these triplets. Higher triplets like T_4_ at the equilibrium geometry appear at significantly higher energies. The latter can be directly related to the position and orientation of the carbonyl group where the transition orbitals do not show the mixing with aromatic orbitals as in 9AA, and therefore remain at energies well above the ππ* states (including S_1_,T_2_ and T_3_ for 2AA). All of the above are consistent with the much longer ^1^L_a_ lifetimes (1 ns in cyclohexane and up to 15 ns in methanol) for 2AA in comparison with 9AA.

**Figure 9:**
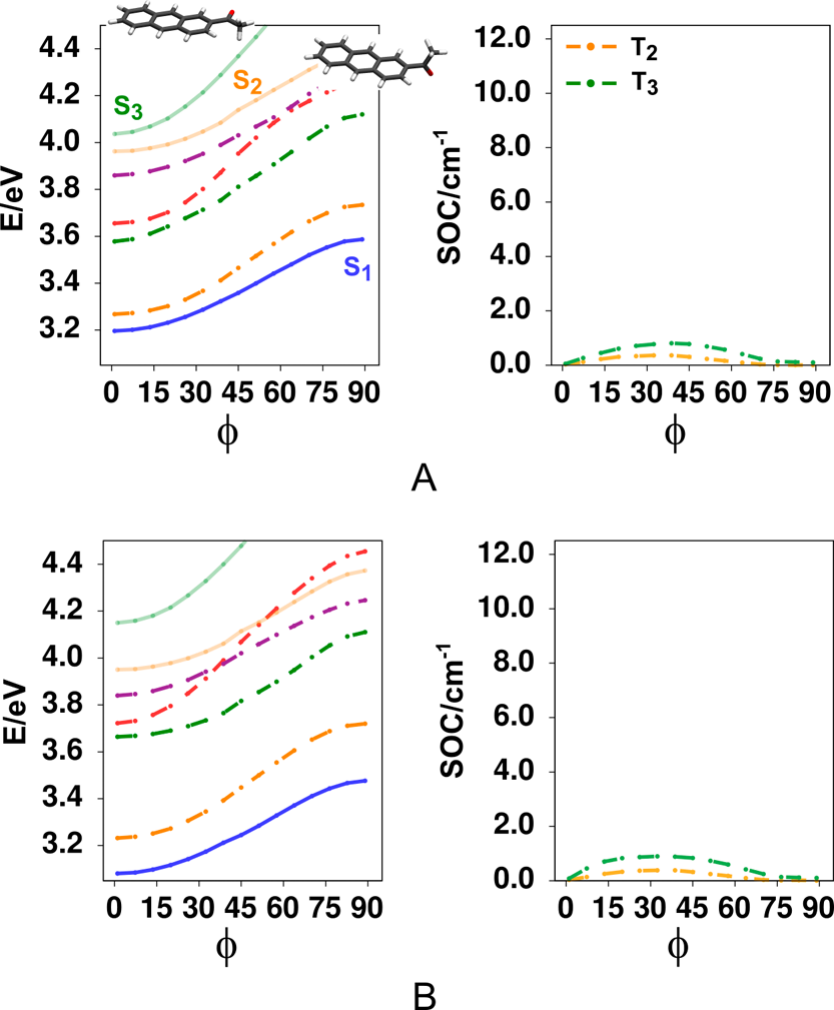
Relaxed scan for the ^1^L_a_ state, as a function of the OCCC dihedral angle, as well as the corresponding spin–orbit coupling values between ^1^L_a_ and the different triplets in 2AA. The energy values are referenced to the minimum of S_0_. (A) gas phase, (B) acetonitrile. The curves in the SOC graph indicate the electronic coupling terms only. As can be seen, the T_4_ and T_5_ couplings are not relevant due to the large energy gap to these states, while for the T_2_ and T_3_ states the couplings remain low (and are zero at the equilibrium geometry) due to the nature of the transitions which are localized at the anthracenic rings.

In summary, exploration by the acetyl substituent in 2AA along the dihedral coordinate is more restricted in this molecule. Additionally, for this molecule the T_2_ and T_3_ states exhibit a pronounced ^3^ππ* character and a minimal ^3^nπ* configurational mixture. This contrasts with what is observed in 9AA, indicating that the substituent position is also a relevant variable for ISC. That is, this difference in configurational mixing when the acetyl group is at position 9, with the respective gain in ^3^nπ* character as the system acquires a tilted geometry is the reason for the higher SOC values observed in 9AA.

## 4 Conclusions

The present investigation contributes to a greater clarity of the photodynamics of deactivation of the S_1_ excited state in aromatic carbonyls by complementing and clarifying previous transient absorption studies and establishing the kind of singlet–triplet couplings present in substituted aromatics where the substituent can display orientations which are slanted with respect to the aromatic plane. In summary, for 9-acetylanthracene (9AA) the S_1_(^1^L_a_) state undergoes at least partial structural relaxation without crossings with other singlets, and remains of emissive ππ* character (^1^L_a_) throughout this path. The torsional motion of the substituent takes place mainly in the subpicosecond time scale in all solvents and is reflected by a shift in the emission spectrum. This is fully consistent with the computational results. ISC takes place after the carbonyl-reorientation as the dominant pathway for the S_1_ state in 9AA with time constants that range from 3.4 ps (cyclohexane), to 25.7 ps (methanol). In at least some of these cases, full vibrational relaxation is unlikely to occur before ISC from comparisons with reported vibrational cooling rates. When the acetyl group corresponds to anthracene position 2 as in the case of 2-acetylanthracene (2AA), the near planarity of the substituent effectively corresponds with reduced couplings with the triplet manifold and much longer fluorescent state lifetime of up to 15 ns. Since in 2AA the carbonyl group remains at a coplanar orientation, the respective ^3^nπ* states lie a significantly higher energies in comparison with 9AA which directly explain the difference between these two systems.

Computational studies with specific TDDFT methods resulting from benchmarking comparisons with the XMS-RASPT2 level of theory achieve a consistent view of the couplings, relative energies within the singlet manifold, and the effects of the carbonyl group orientations and solvent effects, where the 9AA/cyclohexane corresponds to the system with the largest calculated couplings, smallest S_1_–T_2_ energy crossing gaps and fastest ISC rate. The slanted geometry near crossing points between the S_1_ and T_2_ surfaces imply significant spin–orbit couplings. Furthermore, the T_2_ state’s stability is influenced by the oblique geometry, which allows for the respective Kohn–Sham orbitals to be of a mixed nature between those localized at the substituent, and the aromatic anthracenic orbitals. The present studies highlight the importance of the orientation of this kind of substituents, where similar effects have been isolated for nitrated polyaromatic but are related to different electron configurations for the receiver triplet (T_2_). While in nitroaromatics the T_2_ state can entail ^3^ππ* states where the π orbital is associated with a n_Oπ_ character, the T_2_ state in 9AA has a partial nπ* character typical of aromatic carbonyls.

Future studies are required to assess whether the ISC in this kind of systems are also increased by the solute’s vibrational temperature and second order effects, including spin-vibronic couplings which are likely to increase even further these kinds of interaction from motions of the dihedral angle of the substituent orientation and other polyaromatic modes.

## Supplementary Material



## Notes

Excitation spectra, quantum yields comparison with previous measurements, time-resolved convoluted fits parameters, up-conversion results for 9AA in acetonitrile and methanol, comparison of up-conversion traces at different temperatures, TCSPC results, details of computational results including excitation energies for all methods, orbital isosurface images, predicted absorption and emission spectra, and potential energy curves for low frequency modes and SOC values (PDF)
