# Supplementary material for: New Insights into Intersystem Crossing in Substituted Aromatics: Singlet–Triplet Conversion in Carbonyl-Substituted Anthracenes
Source: J Phys Chem B. 2026 Feb 3;130(7):2236–48. doi: 10.1021/acs.jpcb.5c07987 (PMC12926942; doi:10.1021/acs.jpcb.5c07987)
Supplement: Supplementary file 1 [file jp5c07987_si_001.pdf]

SUPPORTING INFORMATION

New Insights into Intersystem Crossing in Substituted Aromatics. Singlet-Triplet Conversion in Carbonyl-substituted Anthracenes

*Cesar A. Guarin[c], Alejandro Larios-Sandoval[a], Michelle Avila-Serna[a], Melissa Bravo-Romero[a], Jesús Jara-Cortés[b], Antonio Resendiz-Pérez[b], and Jorge Peon[a,]**

[a]Universidad Nacional Autónoma de México, Instituto de Química, Ciudad Universitaria, Circuito Exterior, 04510, México

[b]Unidad Académica de Ciencias Básicas e Ingenierías, Universidad Autónoma de Nayarit, Tepic 63155, México

[c]Universidad Autónoma Metropolitana, San Rafael Atlixco, Col-Vicentina, Ciudad de México, 09310, México.

1) Steady-State Section

9AA spectra.

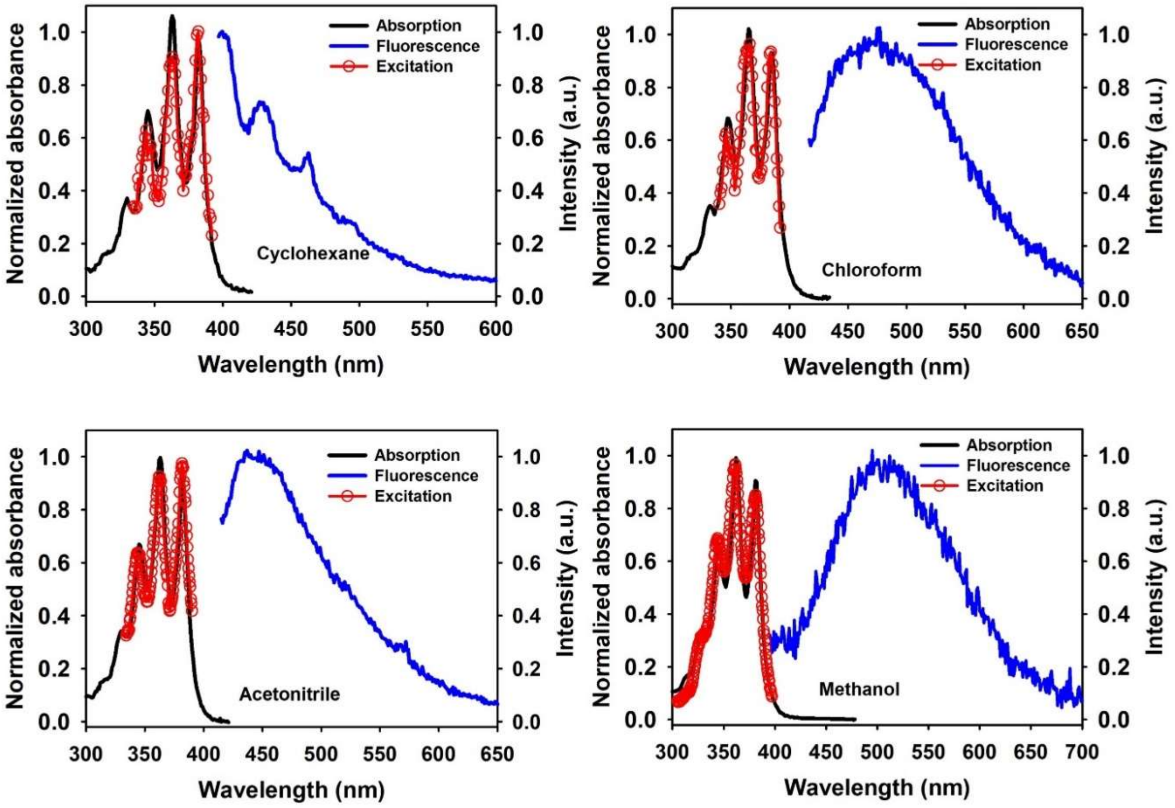

Figure S1. Steady-state spectra for 9AA. The fluorescence excitation spectra of 9AA in cyclohexane, acetonitrile, chloroform, and methanol are shown as red circles. The excitation wavelength for the emission spectra was 360 nm, and the detection wavelength for the excitation spectra was 480 nm.

Comparison of fluorescence quantum yields with previously reported values

Table S1. Comparison of fluorescence quantum yields from the present study with those reported previously

Molecule	Fluorescence Quantum Yield		
	Cyclohexane	Acetonitrile	Methanol
2AA	0.036 ± 0.013 (0.035)[a]	0.53 ± 0.06 (0.49)[a]	0.78 ± 0.02 (0.77)[a]
9AA	4.4×10^{-5} ($<10^{-4}$)[b]	2.0×10^{-4} ---	0.0018 ± 0.0003 (0.0016)[b]

[a]Values taken from reference 1.
[b]Values taken from reference 2.

2) Time-resolved fluorescence section

Table S2. Fit Parameters obtained from the up-conversion transients of the S_1 of 9AA in cyclohexane

λ_{fluor}[nm]	%a_1	τ_1 [ps]	%a_2	τ_2 [ps]
420	99.1±6.1	0.4±0.1	0.9±0.4	3.4±0.6
440	97.7±5.9	0.4±0.1	2.3±0.5	3.4±0.6
450	98.6±4.8	0.4±0.1	1.4±0.5	3.4±0.6
460	95.0±11.4	0.4±0.1	5.0±0.9	3.4±0.6
470	93.0±6.6	0.4±0.1	7.0±0.9	3.4±0.6
490	85.0±7.0	0.4±0.1	15.0±1.0	3.4±0.6
500	76.4±6.2	0.4±0.1	23.6±2.1	3.4±0.6
510	69.1±4.5	0.4±0.1	30.9±1.1	3.4±0.6

Table S3. Fit Parameters obtained from the up-conversion transients of the S_1 state of 9AA in chloroform

λ_{fluor}[nm]	%a_1	τ_1 [ps]	%a_2	τ_2 [ps]	%a_3	τ_3 [ps]
440	49.6±11.3	0.6±0.1	41.1±7.8	2.3±0.4	9.2±2.1	23.8±2.3
460	41.6±10.9	0.6±0.1	48.9±10.2	2.3±0.4	9.5±2.2	23.8±2.3
480	22.5±9.3	0.6±0.1	62.8±10.1	2.3±0.4	14.7±3.1	23.8±2.3
500	-100.0±4.2	0.6±0.1	69.9±9.8	2.3±0.4	30.1±2.4	23.8±2.3
540	-100.0±6.3	0.6±0.1	26.2±7.4	2.3±0.4	73.8±3.3	23.8±2.3
560	-83.9±4.8	0.6±0.1	-16.1±14.5	2.3±0.4	100.0±7.6	23.8±2.3
575	-85.2±3.7	0.6±0.1	-14.8±11.1	2.3±0.4	100.0±6.3	23.8±2.3

Table S4. Fit Parameters obtained from the up-conversion transients of the S_1 state of 9AA in acetonitrile

λ_{fluor}[nm]	%a_1	τ_1 [ps]	%a_2	τ_2 [ps]	%a_3	τ_3 [ps]
420	44.2±5.84	0.3±0.1	54.5±19.6	0.9±0.2	1.2±0.6	8.2±0.8
430	42.4±6.50	0.3±0.1	56.5±22.8	0.9±0.2	1.1±0.4	8.2±0.8
440	19.9±0.83	0.3±0.1	77.1±18.9	0.9±0.2	3.1±0.2	8.2±0.8
460	-100.0±5.12	0.3±0.1	93.5±8.2	0.9±0.2	6.5±0.4	8.2±0.8
490	-100.0±5.65	0.3±0.1	85.2±7.9	0.9±0.2	14.8±0.6	8.2±0.8
510	-100.0±5.44	0.3±0.1	81.2±7.2	0.9±0.2	18.8±0.8	8.2±0.8
530	-100.0±9.38	0.3±0.1	66.6±6.3	0.9±0.2	33.4±5.4	8.2±0.8
540	-100.0±6.75	0.3±0.1	59.5±9.2	0.9±0.2	40.5±6.8	8.2±0.8
560	-100.0±9.79	0.3±0.1	42.8±6.3	0.9±0.2	57.2±9.2	8.2±0.8

Table S5. Fit Parameters obtained from the up-conversion transients of the S_1 state of 9AA in Methanol

λ_{fluor}[nm]	%a_1	τ_1 [ps]	%a_2	τ_2 [ps]	%a_3	τ_3 [ps]
420	83.6±5.1	0.5±0.1	14.1±4.5	2.8±0.3	2.3±1.1	25.7±3
440	81.2±5.3	0.5±0.1	14.1±4.7	2.8±0.3	4.7±1.2	25.7±3
460	63.4±6.2	0.5±0.1	32.3±4.3	2.8±0.3	4.3±1.9	25.7±3
480	29.6±8.9	0.5±0.1	56.3±5.2	2.8±0.3	14.1±2.2	25.7±3
500	-100.0±28.6	0.5±0.1	59.1±7.0	2.8±0.3	40.9±2.6	25.7±3
520	-100.0±32.4	0.5±0.1	32.5±5.8	2.8±0.3	67.5±2.5	25.7±3
540	-100.0±28.2	0.5±0.1	24.1±6.0	2.8±0.3	75.9±2.6	25.7±3
560	-62.7±17.9	0.5±0.1	-37.3±11.9	2.8±0.3	100.0±3.4	25.7±3

Fluorescence decays of the excited state S_1 of 9AA.

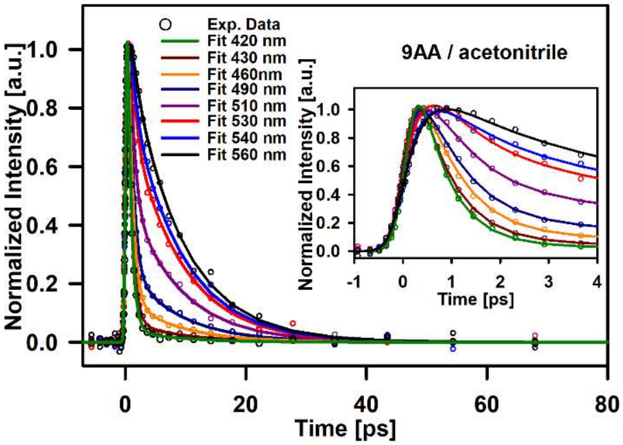

Figure S2. Femtosecond up-conversion results for 9AA in acetonitrile. Inset: The relaxation dynamics in the S_1 state are shown in detail in the time-window from 0 to 4 ps. The inset shows the early evolution of the same traces.

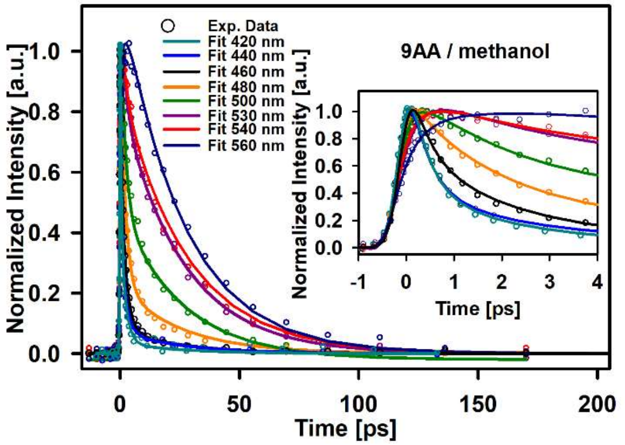

Figure S3. Femtosecond up-conversion results for 9AA in methanol. Inset: The relaxation dynamics in the S_1 state are shown in detail in the time-window from 0 to 4 ps. The inset shows the early evolution of the same traces.

Table S6. Fit parameters obtained from the up-conversion transients of the S_1 of 2AA in cyclohexane

λ_{fluor}[nm]	%a_1	τ_1 [ps]	%a_2	τ_2 [ps]	%a_3	τ_3 [ns]
410	100±6.2	0.3±0.1	6.6±0.8	19.1±2.0	93.4±0.7	1.04±0.02
432	100±23	0.3±0.1	3.3±0.6	19.1±2.0	96.7±0.4	1.04±0.02
450	100±13	0.3±0.1	16.6±1.3	19.1±2.0	83.4±0.7	1.04±0.02
470	91.6±6.3	0.3±0.1	8.4±1.0	19.1±2.0	100±0.6	1.04±0.02
480	100±6.8	0.3±0.1	20.0±1.3	19.1±2.0	80.0±0.8	1.04±0.02
490	98.3±7.6	0.3±0.1	1.7±0.1	19.1±2.0	100±0.5	1.04±0.02
510	100±16	0.3±0.1	1.2±0.7	19.1±2.0	98.8±1.7	1.04±0.02
530	100±10	0.3±0.1	15.8±1.7	19.1±2.0	84.2±1.0	1.04±0.02

Table S7. Fit parameters obtained from the up-conversion transients of the S_1 of 2AA in acetonitrile

λ_{fluor}[nm]	%a_1	τ_1 [ps]	%a_2	τ_2 [ps]	%a_3	τ_3 [ns]
432	11.9±1.6	0.5±0.1	7.1±1.1	21.8±1.4	81.0±0.6	11.32±0.74
456	100±7.4	0.5±0.1	10.7±0.6	21.8±1.4	89.3±0.3	11.32±0.74
470	71.7±8.7	0.5±0.1	28.3±2.2	21.8±1.4	100±0.3	11.32±0.74
480	100±3.4	0.5±0.1	1.7±0.3	21.8±1.4	98.3±0.3	11.32±0.74
490	70.4±6.4	0.5±0.1	29.6±1.7	21.8±1.4	100±0.4	11.32±0.74
510	60.3±5.5	0.5±0.1	39.7±1.3	21.8±1.4	100±0.4	11.32±0.74
530	62.8±5.2	0.5±0.1	37.2±1.1	21.8±1.4	100±0.4	11.32±0.74

Table S8. Fit parameters obtained from the up-conversion transients of the S_1 of 2AA in methanol

λ_{fluor}[nm]	%a_1	τ_1 [ps]	%a_2	τ_2 [ps]	%a_3	τ_3 [ns]
430	24.9±4.5	4.9±0.7	63.7±1.7	16.7±1.6	11.3±2.2	15.02±1.3
450	100±11	4.9±0.7	79.6±1.9	16.7±1.6	20.4±1.7	15.02±1.3
490	100±5	4.9±0.7	39.8±4.3	16.7±1.6	60.2±0.7	15.02±1.3
510	100±5	4.9±0.7	26.8±5.2	16.7±1.6	73.2±0.8	15.02±1.3
556	28.7±3.1	4.9±0.7	71.3±3.3	16.7±1.6	100±0.4	15.02±1.3
580	7.0±1.2	4.9±0.7	93.0±3.3	16.7±1.6	100±0.7	15.02±1.3
595	1.6±0.9	4.9±0.7	98.4±1.4	16.7±1.6	100±0.3	15.02±1.3

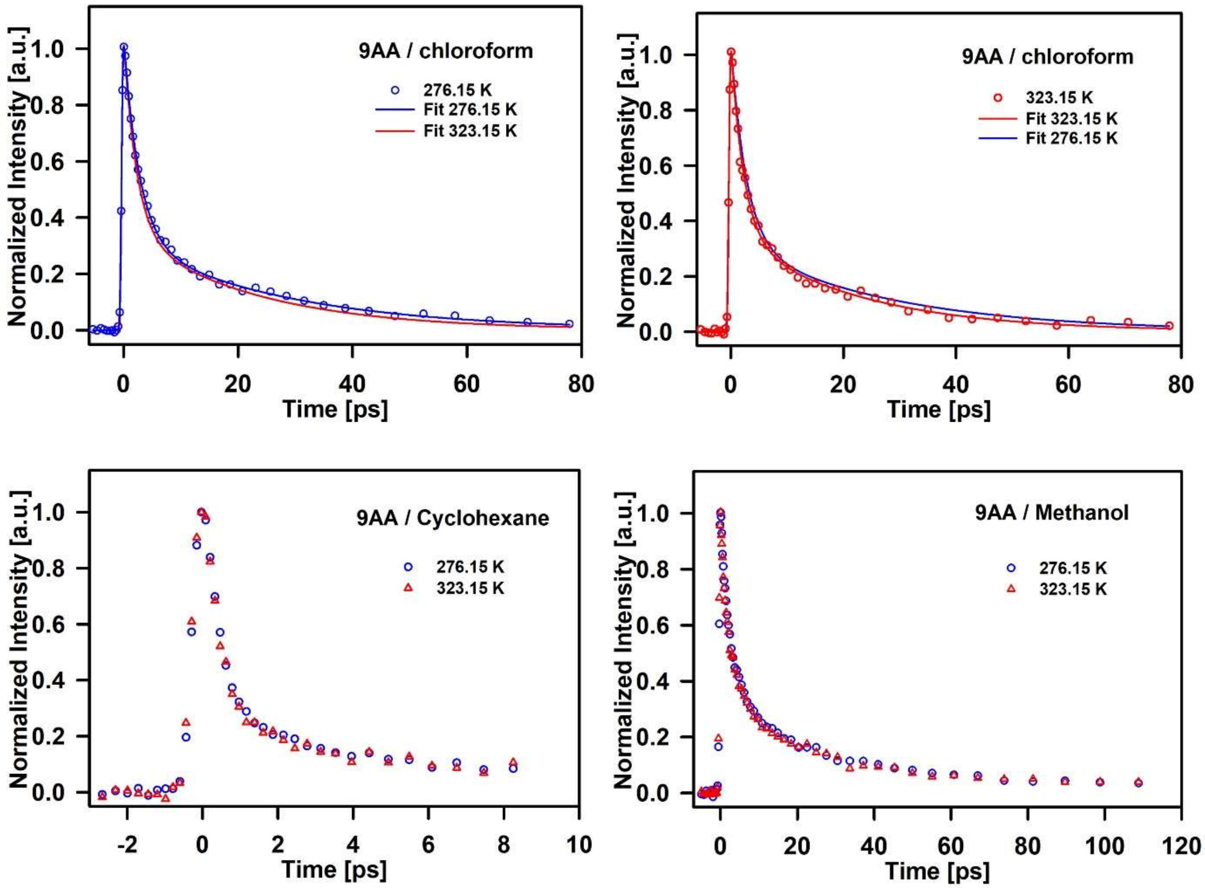

Figure S4. Femtosecond Fluorescence Up-Conversion Measurements of 9AA at 276.15 and 323.15 K.

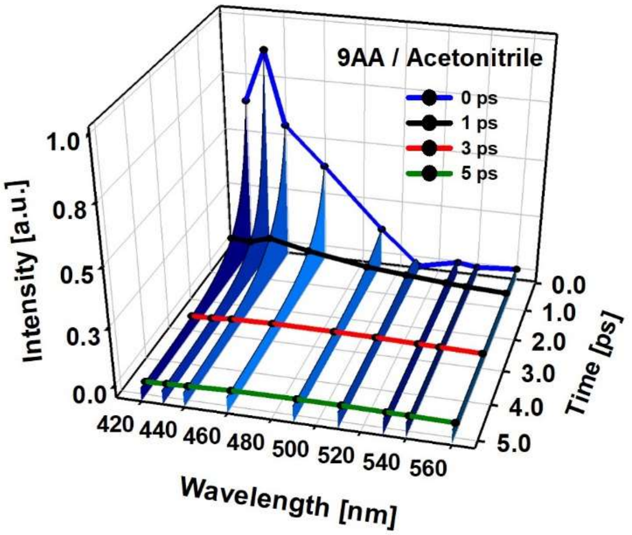

Figure S5. Time-resolved emission spectra reconstructed from the femtosecond up-conversion transient fits for 9AA in acetonitrile.

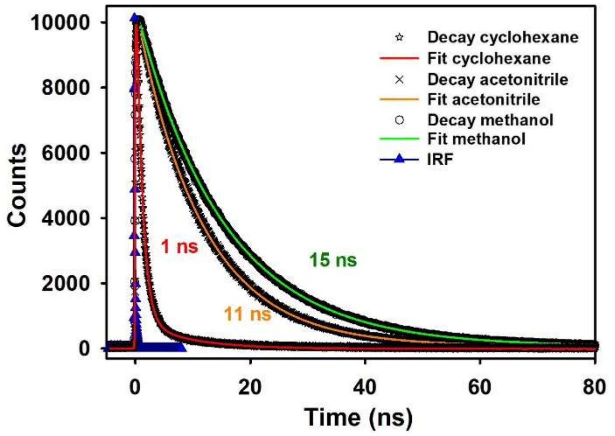

Figure S6. TCSPC results for 2AA in cyclohexane, acetonitrile and methanol.

3) Computational Results

Details on the selection of Time Dependent Functional methods

The comparison between the FLR method and the TDA approximation reveals directly how each method provides better results respectively for the singlet and triplet manifolds. This was evaluated in the benchmarking process through comparisons between the TDDFT gas-phase calculations and the XMS-RASPT2 method in the gas phase, as well as with comparisons with experimental data (absorption and emission spectra from the present study in cyclohexane, and the phosphorescence transitions for 9AA from the literature (see below)). The results are included from **Figures S7** to **S15** and **Tables S9** to **S14**. Refer to the main text for a general description of the results. **Tables S9** and **S10** include transition energies from the gas-phase XMS-RASPT2 calculations for 9AA and 2AA respectively at the S_0 optimized geometries, while **Figures S7** and **S8** include depictions of the relevant orbitals. **Figures S13** to **S14** include the transition energies for the low-lying excited states for 9AA and 2AA (details in the table's headers). The CAM-B3LYP method provides the best overall performance with respect to the reference calculation, with a 0.26 eV difference for the S_1 energy (FLR), and 0.13 eV for the T_1 energy (TDA method).

The choice of the CAM-B3LYP is supported by comparisons with experimental data. For example, for 9AA/cyclohexane, the FLR-CAM-B3LYP transition energy for absorption (3.49 eV) compares well with the experimental maximum (3.42 eV). The energy for the first triplet phosphorescence emission transition (T_1) from the literature (1.825 eV, EPA matrix, 77 K),[3] also agrees with that calculated with appropriate TDDFT method. Specifically, TDA-CAM-B3LYP predicts 1.82 eV in cyclohexane at the T_1 (^3L_a) geometry. In addition, **Figure**

S9 shows simulated absorption and fluorescence spectra of 9AA and 2AA. These profiles were obtained from 500 structures sampled from a Wigner distribution obtained from the normal modes of the S_0 and S_1 (^1L_a) optimized geometries respectively.[4-6] These distributions are to be compared with the maxima of the observed spectra (**Table S11**). As can be seen, the absorption spectrum maxima are within 4 nm compared to that in cyclohexane (note the minimal effect of solvents in the absorption spectrum in **Figure 1**), while the emission spectra is within 20 nm. The larger difference in the emission case is likely to be due to the fact that the total emissions from 9AA measured in cyclohexane include a fraction of pre-relaxed configurations from the short lifetime, while the calculations correspond to the fully relaxed state. On the other hand, for 2AA, which's S_1 state is much longer lived (so the steady state spectrum fully corresponds to relaxed geometries) there is excellent match as both the absorption and emission spectra are within 1 nm (**Table S11**).

Another test for the accuracy of the selected TDDFT method was determined from vibro-electronic spectra constructed following Fermi's golden rule, considering Herzberg-Teller couplings at the geometries of the S_0 and S_1 (^1L_a) states.[7] Here we make use of the fact that the absorption and emission spectra in cyclohexane show clear vibroelectronic structure. The calculated vibro-electronic spectra are shown in **Figure S10**. As can be seen, there is an appropriate agreement with the experimental absorption spectra which are practically insensitive to the solvent polarity. When the selected TDDFT computed spectra are compared with the cyclohexane solutions, the coincidence for 9AA is within 15 nm for both the $0\rightarrow0$ and $0\rightarrow1$ transitions (**Table S12**). The emission progression for 9AA is also in agreement with that observed in cyclohexane, with a 3 nm difference for the $0\leftarrow0$ transition and 5 nm for the $1\leftarrow0$ transition. These transition energies and the overall shape of the vibro-electronic

spectrum are consistent with those observed for 9AA in a 77 K EPA matrix, where the vibro-electronic peaks appear systematically at 10 nm shorter wavelengths in comparison with the calculated ones (**Table S12**). This is expected as the 77 K spectra represents structures where the equilibration of the carbonyl geometry is halted, therefore giving slightly shifted peaks.[8] With regard to the calculations of triplet states, the T_1 states of 9AA has been assigned as a ππ* transition from spectroscopic data, consistent with the present calculations.[3] In addition, the 0←0 (and maximum) phosphorescence transition for 9AA has been reported corresponding to a value of 14720 cm^{-1} (1.825 eV, 679.3 nm EPA matrix, 77 K), which is within a 2% error in comparison with the 1.85 eV (14920 cm^{-1}, 670.2 nm) T_1 (^3L_a) energy at this state's equilibrium geometry from the calculations.[3] The comparisons between the experimental transitions and the TDDFT calculations are even better for 2AA, with only an up to 3 nm difference for absorption and 4 nm for the emissions.

Table S9. Excitation energies and oscillator strengths (*f*) for the low-lying singlet and triplet states of 9-acetylanthracene (9AA), evaluated at the S_0 equilibrium geometry with the XMS-RASPT2/MA-def2-SVP level of theory. The RAS spaces comprise 6 (RAS-1), 6 (RAS-2) and 5 (RAS-3) orbitals, respectively, as well as a total of 18 electrons. Information on the main determinants involved in the description of the electronic states is also given.

State	Character	ΔE [eV]	*f*	Determinant	Coefficient
T_1	^3L_a	2.039	--	22222222uu0000000	-0.849
S_1	^1L_a	3.304	0.205	22222222ud0000000	0.868
T_2	^3L_b	3.573	--	22222222u00u00000	-0.563
				222222u22u0000000	0.626
S_2	^1L_b	3.681	0.004	22222222u00d00000	-0.575
				222222u22d0000000	-0.619
T_3	$^3n\pi^*$	3.977	--	2222222u20u000000	-0.808
S_3	$^1n\pi^*$	4.022	0.000	2222222u20d000000	-0.834
T_4	$^3\pi\pi^*$	4.613	--	222222u2200u00000	-0.829

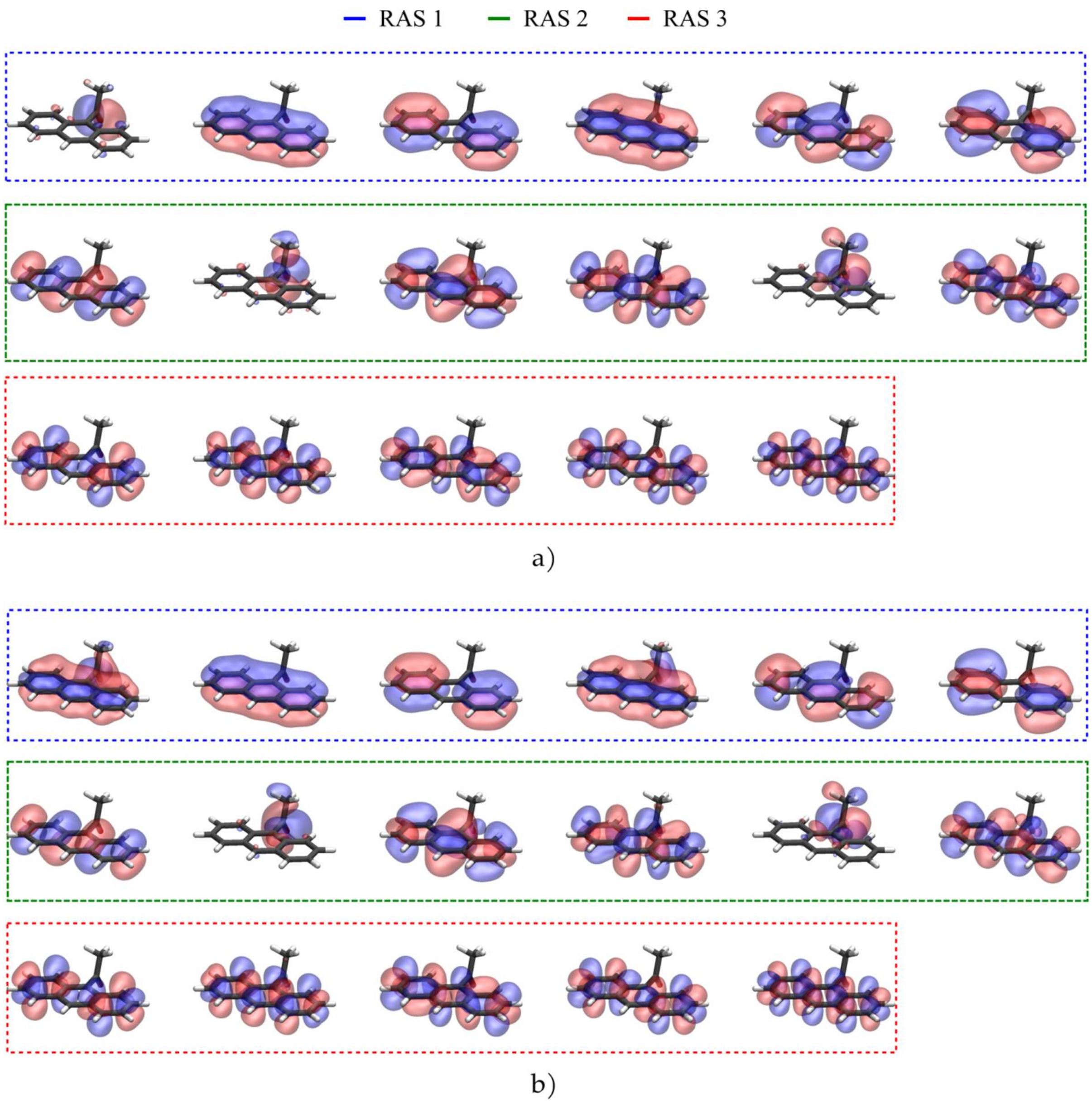

Figure S7 Isosurface (|0.02|) of the molecular orbitals involved in the expansion of the (18e,17o) RASSCF/MA-def2-SVP wave function for the 9-acetylanthracene molecule (9AA). a) Correspond to the (state averaged) orbitals for the singlet manifold, and b) to the triplet manifold. The spaces RAS-1, RAS-2 and RAS-3 involve 6, 6 and 5 orbitals, respectively.

Table S10. Excitation energies and oscillator strengths (*f*) for the low-lying singlet and triplet states of 2-acetylanthracene (2AA), evaluated at the S_0 equilibrium geometry with the XMS-RASPT2/MA-def2-SVP level of theory. The RAS spaces comprise 6 (RAS-1), 6 (RAS-2) and 5 (RAS-3) orbitals, respectively, as well as a total of 18 electrons. Information on the main determinants involved in the description of the electronic states is also given.

State	Character	ΔE [eV]	*f*	Determinant	Coefficient
T_1	^3L_a	2.011	--	22222222uu0000000	0.550
				22222222u0u000000	-0.597
S_1	^1L_a	3.268	0.072	22222222ud0000000	0.794
T_2	$^3n\pi^*$	3.218	--	222222u22u0000000	-0.728
				222222u2200u00000	-0.414
T_3	^3L_b	3.375	--	22222222uu0000000	-0.295
				22222222u0u000000	-0.525
				2222222u2u0000000	0.530
S_2	$^1n\pi^*$	3.535	0.000	222222u22d0000000	-0.670
				222222u220d000000	-0.396
				222222u2200d00000	-0.276
S_3	^1L_b	3.619	0.014	22222222u0d000000	0.526
				22222222u00d00000	-0.282
				2222222u2d0000000	0.528
T_4	$^3\pi\pi^*$	4.340	--	22222222uu0000000	-0.302
				2222222u2u0000000	-0.521
				2222222u20u000000	-0.428

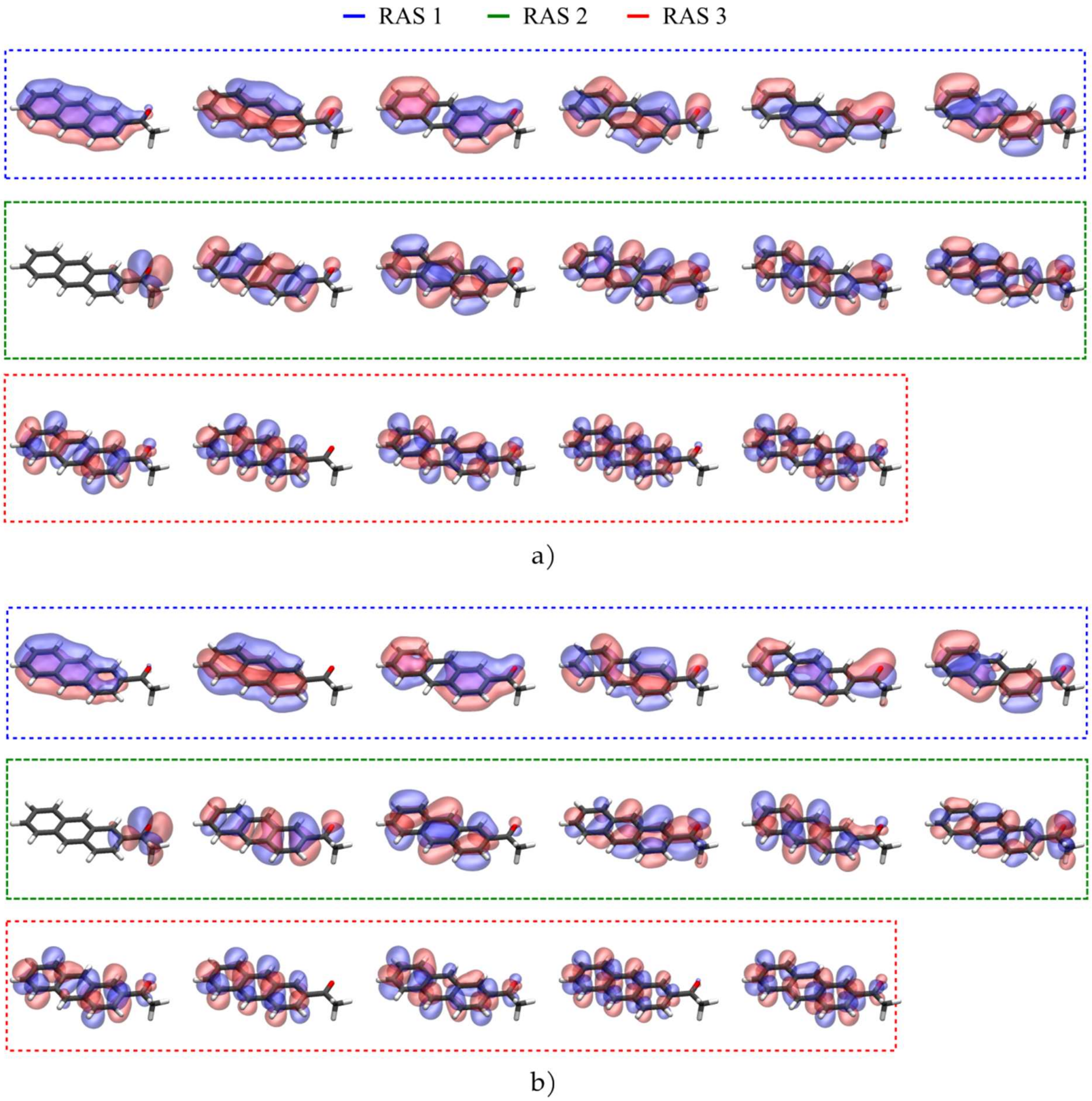

Figure S8. Isosurface (|0.02|) of the molecular orbitals involved in the expansion of the (18e,17o) RASSCF/MA-def2-SVP wave function for the 2-acetylanthracene molecule. a) Correspond to the (state averaged) orbitals for the singlet manifold, and b) to the triplet manifold. The spaces RAS-1, RAS-2 and RAS-3 involve 6, 6 and 5 orbitals, respectively.

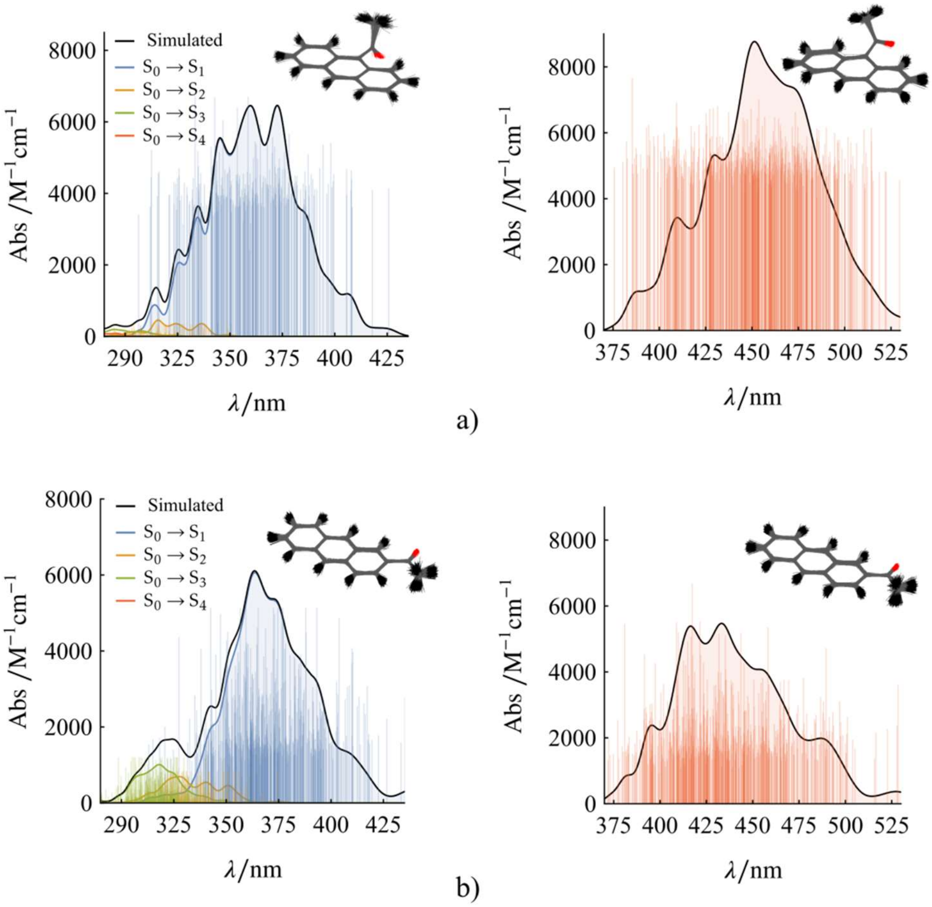

Figure S9. Simulated gas-phase electronic absorption (left) and emission spectra (right) using the selected TDDFT method for a) 9-acetylanthracene (9AA), and b) 2-acetylanthracene (2AA). 500 structures were sampled from the Wigner distribution, using information from the normal modes associated with the minima of S_0 and S_1(^1L_a). For each geometry, the stick spectra are convoluted with Gaussian functions; subsequently, all spectra are averaged. The molecular structures provide a graphical depiction of the sampled nuclear configuration space. The vertical axis on the emission curves (red lines) are proportional to the $S_1 \rightarrow S_0$ oscillator strengths.

Table S11. Comparison between CAM-B3LYP/MA-def2-SVP simulated spectra of 9AA and 2AA with the maxima observed for these molecules in cyclohexane solution

Molecule	Condition	$\lambda_{Absorption}$ (nm)	$\lambda_{Emission}$ (nm)
9AA	Exp. (Cyclohexane)	363	426
	Calc. (gas)	367	448
2AA	Exp. (Cyclohexane)	358	430
	Calc. (gas)	357	431

Table S12. Comparison between the theoretical vibro-electronic spectra evaluated at the TDDFT CAM-B3LYP/MA-def2-SVP level of theory and experimental data for 9AA and 2AA. The calculations were performed within the framework of the Fermi golden rule, considering Herzberg–Teller couplings, based on information from the calculations of S_0 and $S_1(^1L_a)$ vibrational frequencies.

Molecule	Condition	Transition			
		0→0	0→1	0←0	1←0
		$\lambda_{Absorption}$ (nm)		$\lambda_{Emission}$ (nm)	
9AA	Exp. (Cyclohexane)	382	363	400	426
	Exp. (EPA 77 K)[a]	--	--	386	408
	Calc. (gas)	396	373	397	421
	Exp. (Cyclohexane)	400	378	400	430
2AA	Calc. (gas)	403	380	404	434

[a]From reference 8

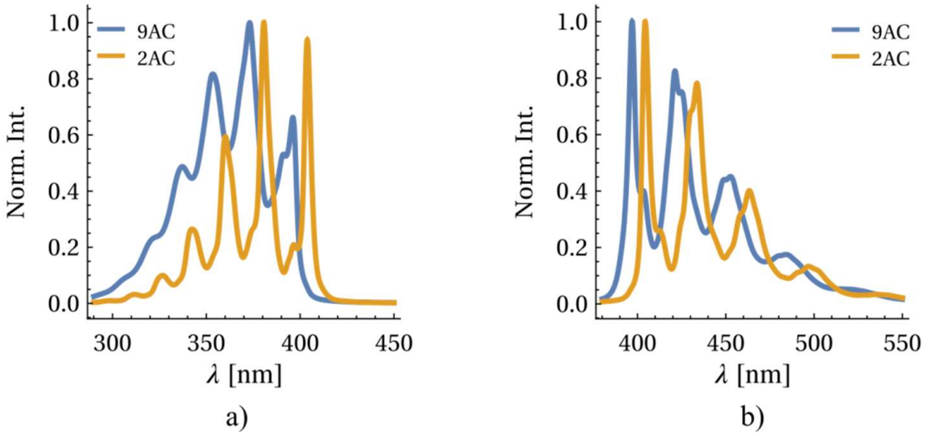

Figure S10. Simulated electronic a) absorption and b) emission spectra for 9-acetylanthracene (9AA), and 2-acetylanthracene (2AA). The spectra were evaluated using Fermi's golden rule approach, considering Herzberg–Teller couplings, based on information from the calculations of S_0 and $S_1(^1L_a)$ vibrational frequencies.

Table S13. Excitation energies (eV) for the low-lying singlet and triplet states of 9-acetylanthracene (9AA), evaluated at the S_0 equilibrium geometry in the gas-phase with the MA-def2-SVP basis set and several density functionals under the full linear-response (FLR) TDDFT approach. The number in parentheses refers to the percentage of fixed Hatree-Fock exchange included in the DFT functional, except for ωB97X, which refers to short-range exchange. The calculations were carried out with the Orca quantum chemistry program.

State	DFT Functionals									
	BLYP	BLYP	TPSS	TPSSh (10%)	B3LYP (20%)	PBE0 (25%)	TPSS0 (25%)	BHHLYP (25%)	ωB97X (15.77%)	CAM-B3LYP (35%)
$1^1\pi\pi^*$	2.78	2.79	2.84	3.04	3.15	3.24	3.29	3.58	3.76	3.56
$2^1\pi\pi^*$	3.02	3.09	3.30	3.64	3.77	3.90	3.95	4.17	4.14	4.05
$3^1\pi\pi^*$	3.49	3.54	3.60	3.77	3.86	4.05	4.15	5.01	5.24	4.79
$4^1\pi\pi^*$	3.75	3.80	3.89	4.23	4.44	4.64	4.72	5.23	5.33	5.17
$5^1\pi\pi^*$	4.19	4.30	4.43	4.71	4.78	4.92	5.00	5.43	5.50	5.36
$1^1n\pi^*$	3.08	3.01	3.11	3.61	4.11	4.18	4.26	4.51	4.30	4.28
$2^1n\pi^*$	3.99	3.99	4.11	4.28	4.29	4.44	4.63	5.66	5.58	5.13
$1^3\pi\pi^*$	1.79	1.77	1.70	1.65	1.74	1.61	1.48	1.17	1.57	1.61
$2^3\pi\pi^*$	2.97	3.03	3.15	3.15	3.23	3.17	3.10	3.03	3.25	3.22
$3^3\pi\pi^*$	3.19	3.21	3.16	3.38	3.43	3.49	3.51	3.66	3.75	3.65
$4^3\pi\pi^*$	3.22	3.25	3.28	3.53	3.64	3.71	3.75	3.93	3.92	3.84
$5^3\pi\pi^*$	3.37	3.40	3.48	3.62	3.89	3.90	3.86	3.96	4.08	4.05
$6^3\pi\pi^*$	3.74	3.78	3.89	4.09	4.33	4.38	4.36	4.45	4.59	4.50
$1^3n\pi^*$	2.99	2.90	2.95	3.30	3.54	3.51	3.55	3.83	3.71	3.66
$2^3n\pi^*$	3.44	3.42	3.55	3.76	3.96	4.13	4.25	0.00	5.44	5.03

Table S14: Excitation energies (eV) for the low-lying singlet and triplet states of 9-acetylanthracene (9AA), evaluated at the S_0 equilibrium geometry with the MA-def2-SVP basis set and several density functionals under the TDDFT approach using the Tamm–Dancoff approximation (TDA). The number in parentheses refers to the percentage of fixed Hatree-Fock exchange included in the DFT functional, except for ωB97X, which refers to short-range exchange. The calculations were carried out with the Orca quantum chemistry program.

State	DFT Functionals									
	BLYP	BLYP	TPSS	TPSSh (10%)	B3LYP (20%)	PBE0 (25%)	TPSS0 (25%)	BHHLYP (25%)	ωB97X (15.77%)	CAM-B3LYP (35%)
$1^1\pi\pi^*$	2.964	3.067	3.031	3.253	3.372	3.467	3.511	3.816	4.033	3.814
$2^1\pi\pi^*$	3.017	3.114	3.333	3.651	3.780	3.916	3.972	4.232	4.236	4.110
$3^1\pi\pi^*$	3.496	3.546	3.612	3.786	3.874	4.067	4.168	5.030	5.261	4.799
$4^1\pi\pi^*$	3.757	3.802	3.902	4.240	4.450	4.647	4.734	5.413	5.615	5.349
$5^1\pi\pi^*$	4.190	4.234	4.447	4.842	4.965	5.100	5.174	5.430	5.871	5.396
$1^1n\pi^*$	3.117	2.938	3.122	3.616	4.118	4.184	4.268	4.564	4.333	4.307
$2^1n\pi^*$	4.007	4.006	4.118	4.292	4.305	4.456	4.641	5.669	5.547	5.155
$1^3\pi\pi^*$	1.894	1.890	1.884	1.942	2.018	2.010	2.013	2.131	2.282	2.173
$2^3\pi\pi^*$	2.971	3.036	3.164	3.393	3.426	3.435	3.447	3.521	3.616	3.535
$3^3\pi\pi^*$	3.239	3.274	3.305	3.425	3.478	3.544	3.585	3.759	3.835	3.711
$4^3\pi\pi^*$	3.290	3.309	3.334	3.546	3.671	3.748	3.805	4.037	4.061	3.929
$5^3\pi\pi^*$	3.380	3.411	3.491	3.642	3.908	4.017	4.034	4.289	4.340	4.282
$6^3\pi\pi^*$	3.739	3.781	3.896	4.219	4.252	4.386	4.568	4.698	4.786	4.679
$1^3n\pi^*$	2.994	2.901	2.968	3.330	3.580	3.570	3.639	3.945	3.762	3.719
$2^3n\pi^*$	3.471	3.449	3.597	3.818	4.013	4.170	4.324		5.465	5.044

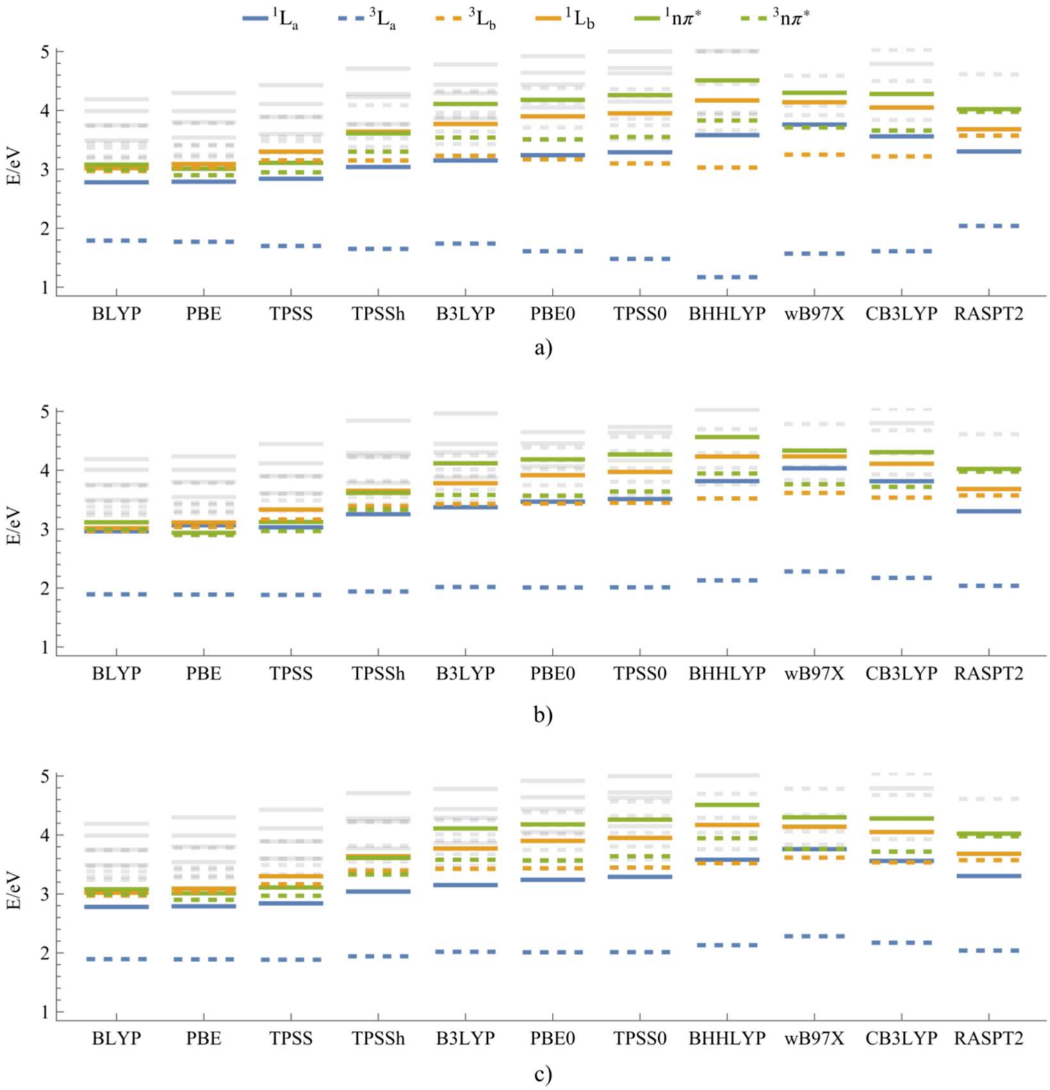

Figure S11. Position of the low-lying singlet (full lines) and triplet (dashed lines) electronic states evaluated at the S_0 equilibrium geometry in the gas phase for 9-acetylanthracene (9AA). The energies were evaluated using different functionals and the MA-def2-SVP basis set and are compared with those obtained using XMS-RASPT2. a) Full-linear response (FLR), b) Tamm-Dancoff approximation (TDA), c) FLR for singlets and TDA for triplets.

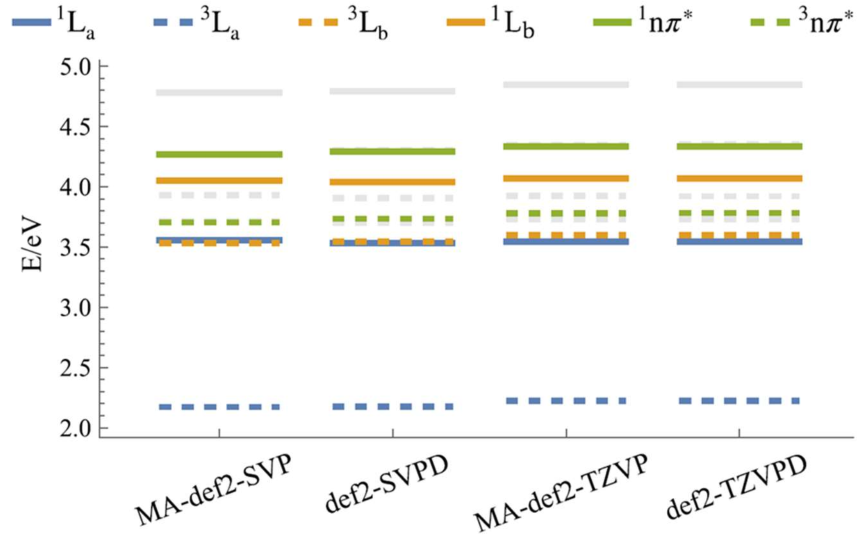

Figure S12. Comparison of the use of different basis sets on the excited state energies of 9-acetylanthracene (9AA gas phase, S_0 geometry) considering the FLR CAM-B3LYP method.

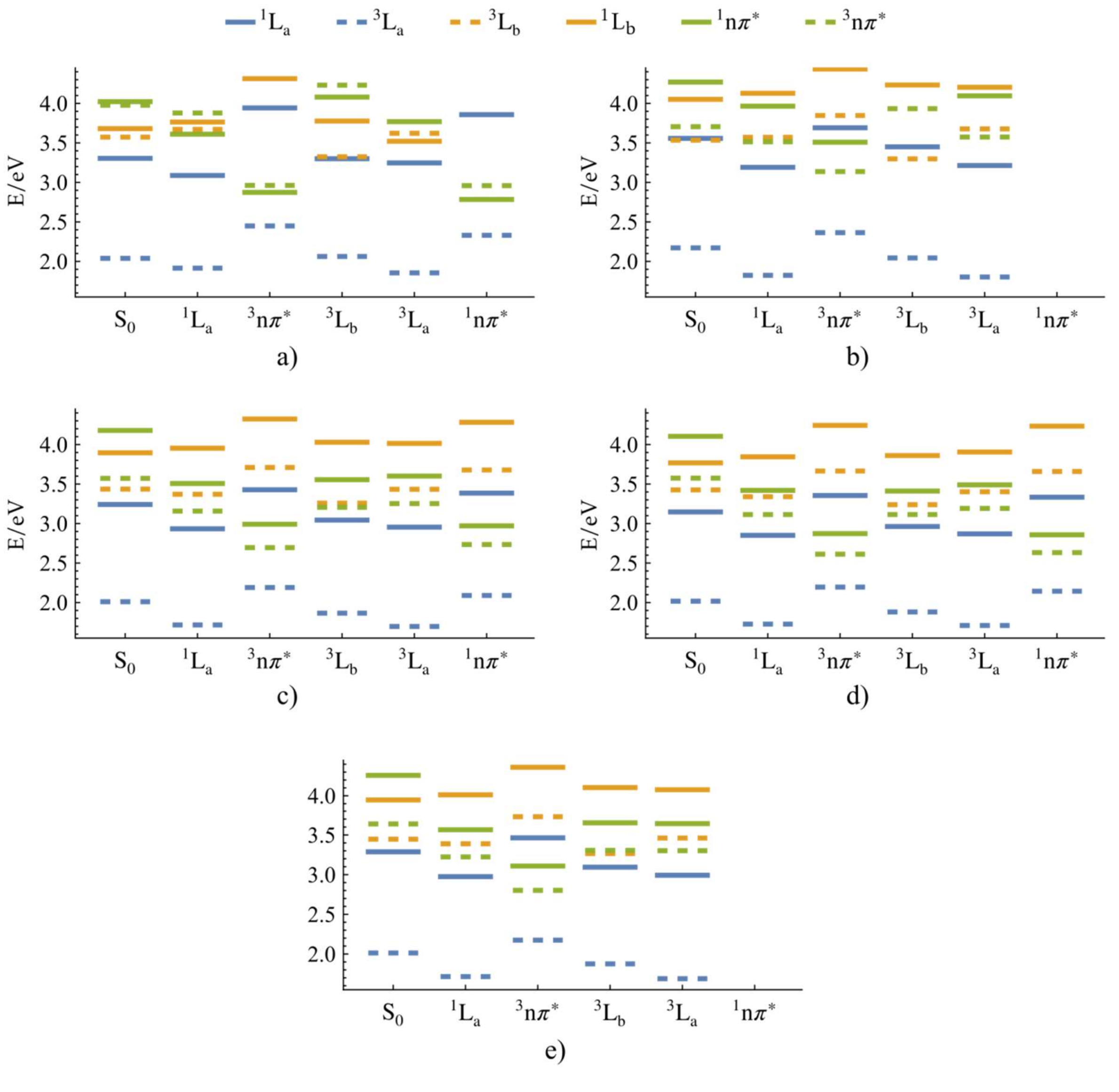

Figure S13. Energies of the low-lying singlet and triplet states, evaluated at selected equilibrium geometries on the excited states potential energy surfaces of 9-acetylanthracene (9AA, gas phase). The calculations were performed using the MA-def2-SVP basis set, employing the method a) XMS-RASPT2, as well with the TDDFT approach using the functionals b) CAM-B3LYP, c) B3LYP, d) PBE0 and e) TPSS0. Singlet and triplet states were obtained with the FLR and TDA approaches, respectively.

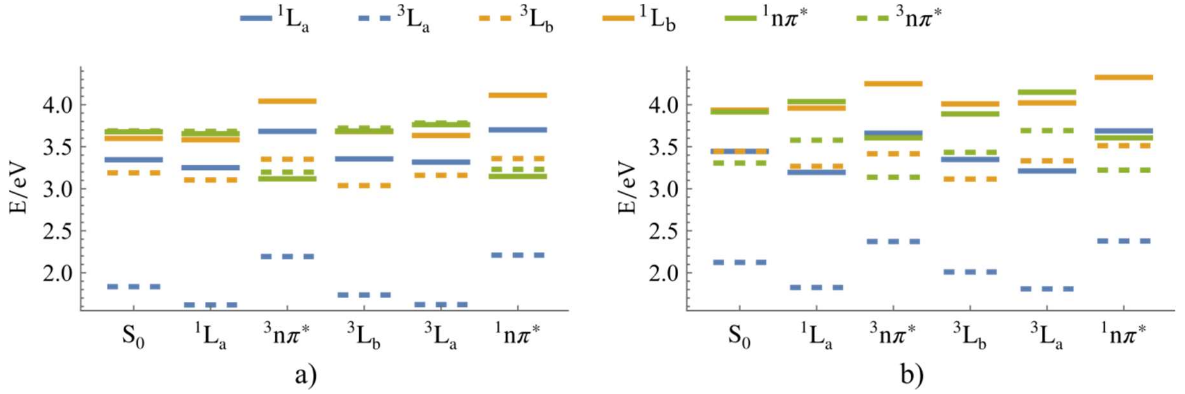

Figure S14. Energies of the low-lying singlet and triplet states, evaluated at selected equilibrium geometries on the excited states potential energy surfaces of 2-acetylanthracene (2AA, gas phase). The calculations were performed using the MA-def2-SVP basis set, employing the method a) XMS-RASPT2, as well with the TDDFT approach using the functional b) CAM-B3LYP. Singlet and triplet states were obtained with the FLR and TDA approaches, respectively.

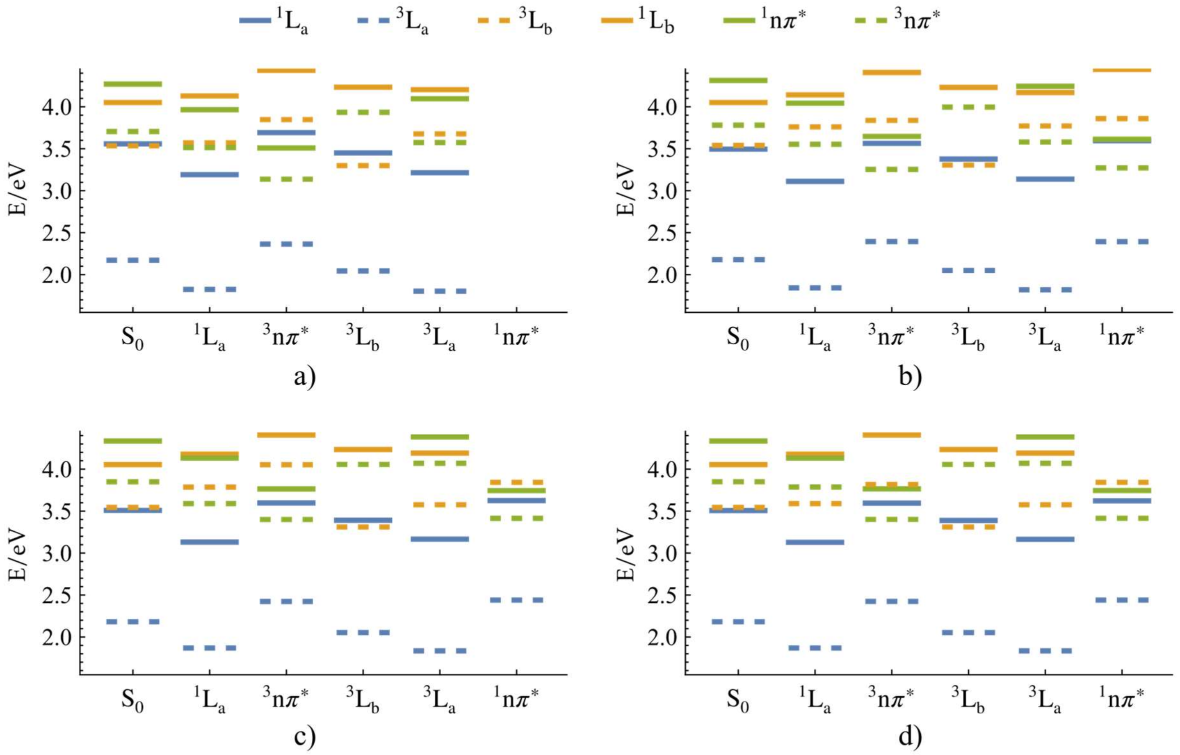

Figure S15. Energies of the low-lying singlet and triplet states, evaluated at selected equilibrium geometries on the excited states potential energy surfaces of 9-acetylanthracene (9AA). The calculations were performed using the CAM-B3LYP/MA-def2-SVP level of theory, taking into consideration the solvent implicitly under the polarizable continuum model approach: a) gas phase, b) cyclohexane, c) methanol, d) acetonitrile. Singlet and triplet states were obtained with the FLR and TDA approaches, respectively.

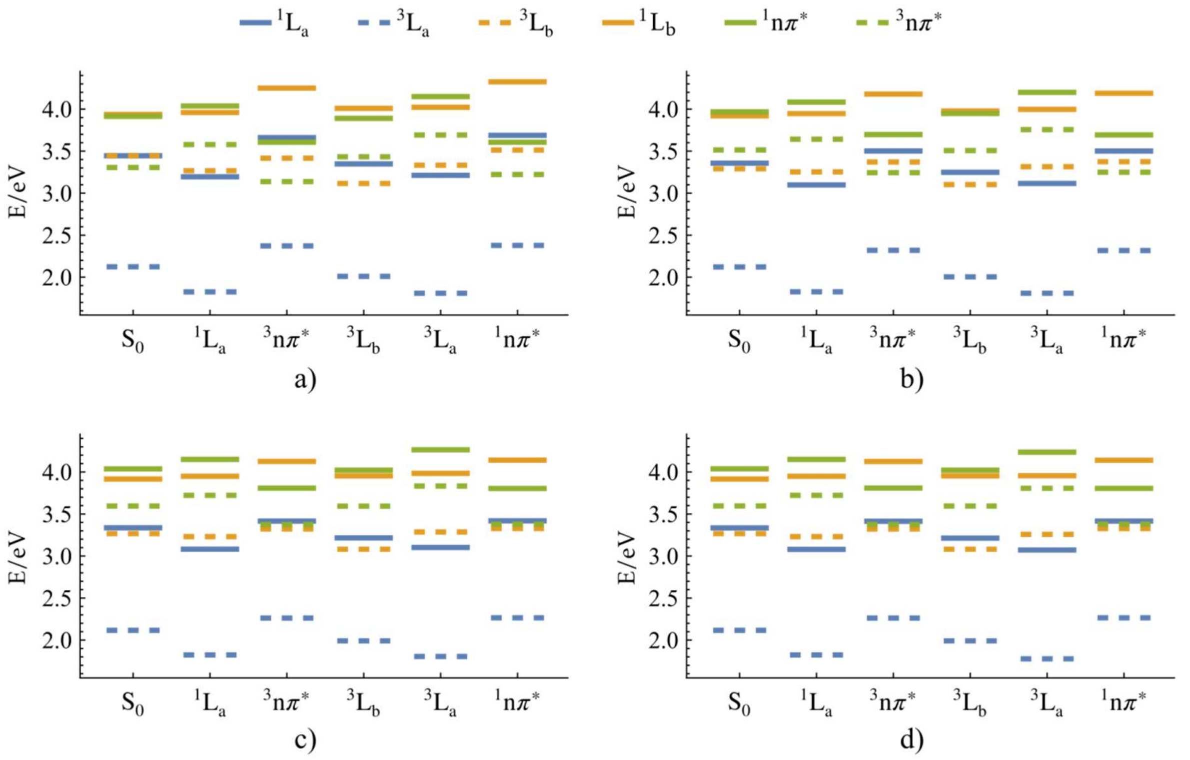

Figure S16. Energies of the low-lying singlet and triplet states, evaluated at selected equilibrium geometries on the excited states potential energy surfaces of 2-acetylanthracene (2AA). The calculations were performed using the CAM-B3LYP/MA-def2-SVP level of theory, taking into consideration the solvent implicitly under the polarizable continuum model approach: a) gas phase, b) cyclohexane, c) methanol, d) acetonitrile. Singlet and triplet states were obtained with the FLR and TDA approaches, respectively.

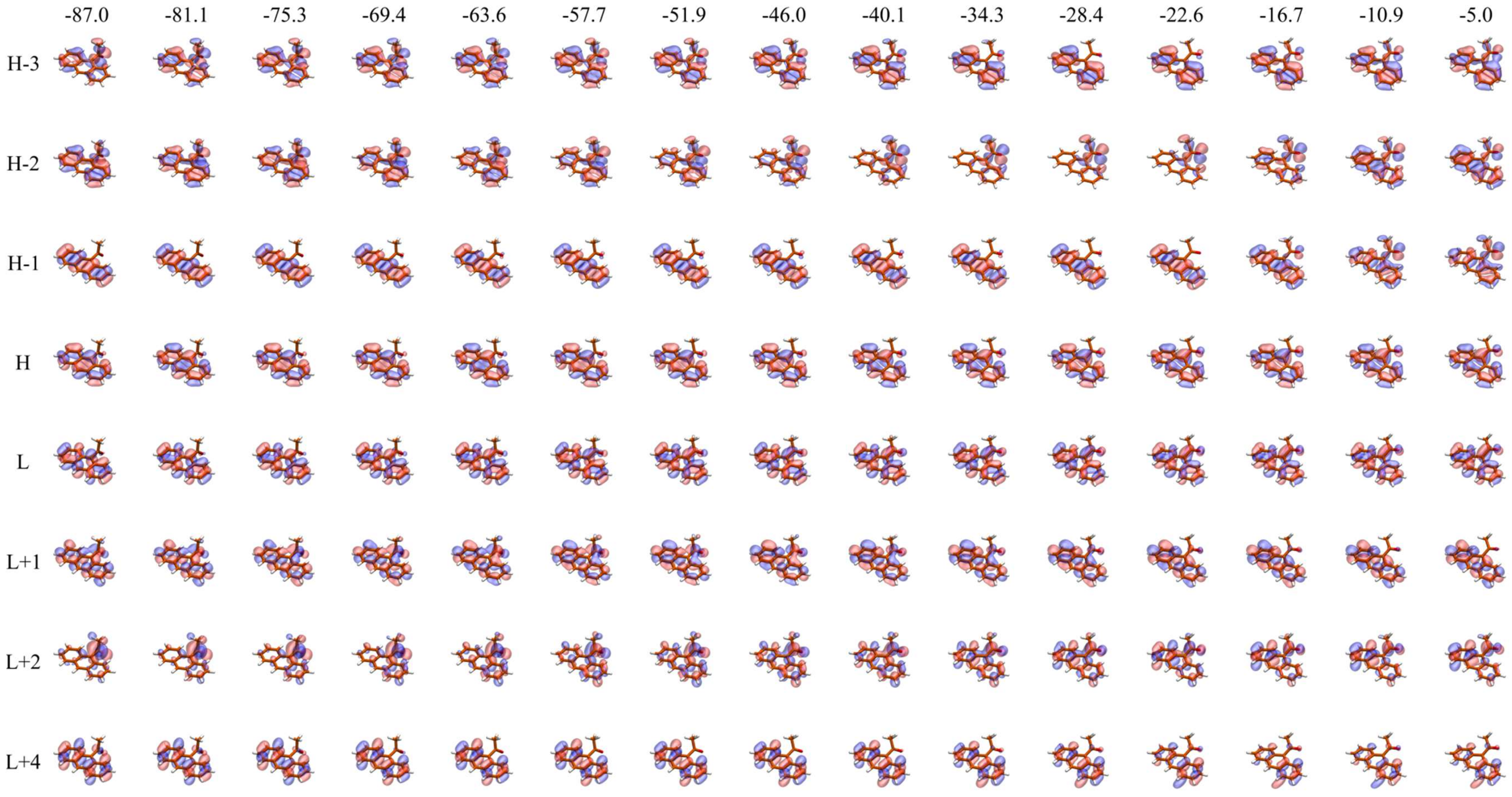

Figure S17. Isosurface (0.03) of the Kohn-Sham molecular orbitals for the relaxed scan of the ^1L_a state, as a function of the OCCC dihedral angle (ϕ). The calculations were performed in the gas phase at the CAM-B3LYP/MA-def2-SVP level of theory.

Table S15. TDDFT amplitudes for the low-energy triplet electronic states of 9AA, along the scan of the dihedral coordinate ϕ. Figure S17 shows the isosurfaces of the respective Kohn-Sham molecular orbitals. The calculations were performed in the gas phase at the CAM-B3LYP/MA-def2-SVP level of theory.

	ϕ=-75.3^0			ϕ=-69.4^0			ϕ=-63.6^0		
**Configuration**	**T_2**	**T_3**	**T_4**	**T_2**	**T_3**	**T_4**	**T_2**	**T_3**	**T_4**
H-3→L	-0.48	--	-0.22	0.50	-0.24	--	-0.51	-0.33	--
H-3→L+1	--	--	0.25	--	0.23	--	--	-0.23	--
H-3→L+2	--	--	0.49	--	0.39	--	--	0.34	--
H-2→L	-0.53	--	0.21	0.51	0.29	--	0.51	-0.37	--
H-2→L+1	--	--	-0.27	--	-0.29	--	--	-0.33	--
H-2→L+2	--	--	-0.49	--	-0.45	--	--	0.46	--
H-1→L	--	0.85	-0.21	--	0.45	-0.77	--	-0.27	0.88
H→L+1	--	0.46	--	--	--	-0.46	--	--	-0.36
H→L+4	0.60	--	--	-0.60	--	--	-0.59	--	--

	ϕ=-46.0^0			ϕ=-40.1^0			ϕ=-34.3^0		
	**T_2**	**T_3**	**T_4**	**T_2**	**T_3**	**T_4**	**T_2**	**T_3**	**T_4**
H-3→L	-0.45	-0.51	-0.25	-0.32	0.62	-0.27	--	-0.70	0.29
H-2→L	0.60	-0.42	--	0.71	0.28	--	-0.78	--	--
H-2→L+1	--	0.29	--	--	--	--	--	--	--
H-2→L+2	--	0.43	--	--	-0.36	--	0.41	--	--
H-1→L	--	-0.33	0.90	--	0.35	0.91	--	-0.35	-0.90
H→L+4	0.48	--	--	-0.36	0.34	--	--	0.41	--

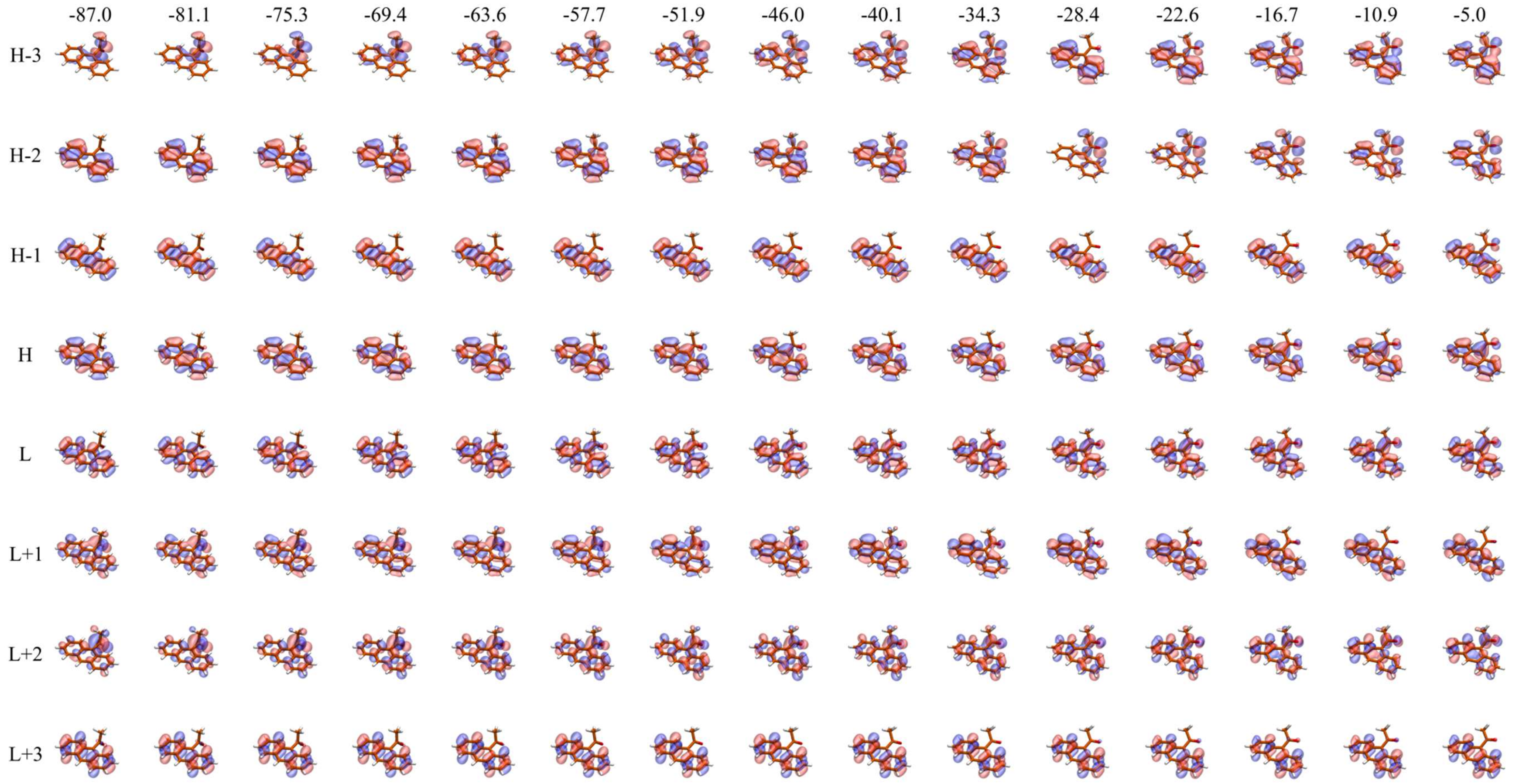

Figure S18. Isosurface (0.03) of the Kohn-Sham molecular orbitals for the relaxed scan of the $^{1}L_{a}$ state, as a function of the OCCC dihedral angle (ϕ). The calculations were performed considering implicit solvation under the PCM approach (Chloroform)

Table S16. TDDFT amplitudes for the low-energy triplet electronic states of 9AA, along the scan of the dihedral coordinate ϕ. Figure S18 shows the isosurfaces of the respective Kohn-Sham molecular orbitals. The calculations were performed considering implicit solvation under the PCM approach (Chloroform) at the CAM-B3LYP/MA-def2-SVP level of theory.

	$\phi=-63.6^0$			$\phi=-57.7^0$			$\phi=-51.8^0$		
**Configuration**	**T_2**	**T_3**	**T_4**	**T_2**	**T_3**	**T_4**	**T_2**	**T_3**	**T_4**
H-3→L	--	--	-0.45	--	-0.38	-0.37	--	-0.47	-0.38
H-3→L+1	--	--	0.43	--	-0.36	--	--	-0.34	--
H-3→L+2	--	--	0.36	--	-0.28	--	--	-0.29	--
H-2→L	0.69	--	--	0.69	--	--	0.69	--	--
H-1→L	--	0.91	--	--	0.63	-0.68	--	0.53	-0.77
H→L+1	--	0.14	-0.52	--	0.20	0.42	--	--	0.31
H→L+3	0.57	--	--	-0.55	--	--	-0.53	--	--

	$\phi=-28.4^0$			$\phi=-22.6^0$			$\phi=-16.7^0$		
	**T_2**	**T_3**	**T_4**	**T_2**	**T_3**	**T_4**	**T_2**	**T_3**	**T_4**
H-3→L	-0.64	--	-0.47	0.29	-0.45	-0.61	0.24	0.30	0.76
H-3→L+2	--	--	--	--	--	--	--	--	--
H-2→L	--	0.81	--	-0.64	-0.54	--	0.86	--	--
H-2→L+1	--	0.22	--	--	--	--	--	--	--
H-2→L+2	--	-0.42	--	--	0.33	--	-0.21	--	--
H-1→L	0.54	--	-0.80	-0.49	0.52	-0.66	--	-0.90	0.39
H→L+3	0.40	--	0.24	-0.27	--	0.31	--	--	-0.35

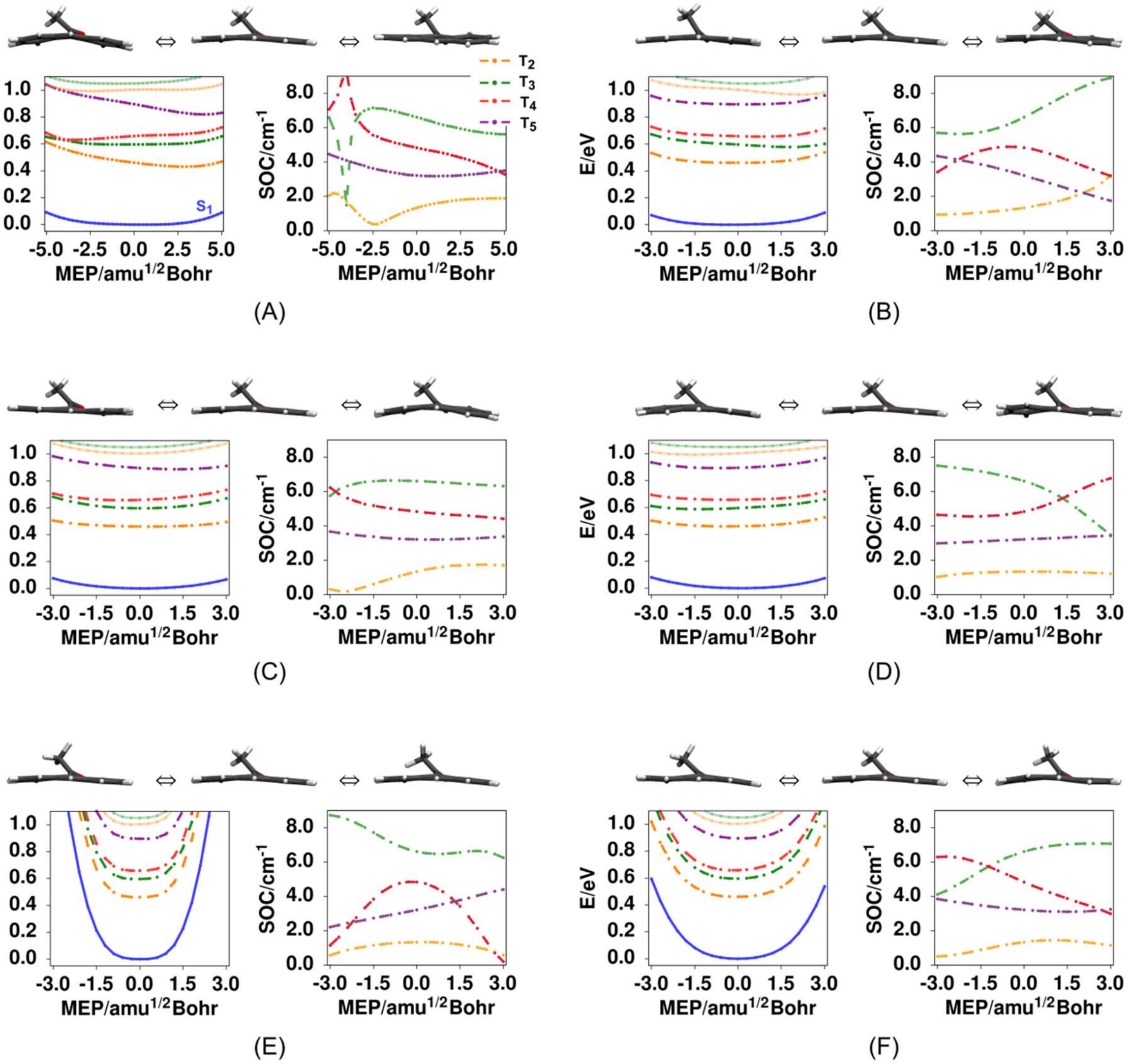

Figure S19. Energies of the low-lying electronic states, as well as the corresponding spin-orbit coupling value, for displacements along the low-frequency vibrational modes A) Q_1 (32.62 cm^{-1}), B) Q_2 (71.68 cm^{-1}), C) Q_3 (97.38 cm^{-1}), D) Q_4 (114.96 cm^{-1}), E) Q_5 (165.97 cm^{-1}) and F) Q_6 (212.98 cm^{-1}) of the ^1L_a state of 9-nitroanthracene. The normal modes were evaluated with at the TDDFT/CAM-B3LYP level of theory (acetonitrile), using the MA-def2-SVP basis set. The energy values are referenced to the minimum of S_1.

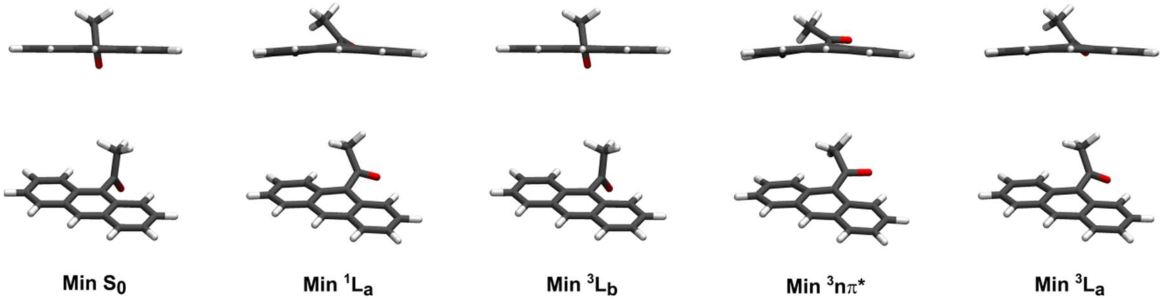

Figure S20. Front and side views of the equilibrium structures for selected low-lying electronic states of 9AA. The calculations correspond to the TDDFT CAM-B3LYP/MA-def2-SVP theory level in gas phase.

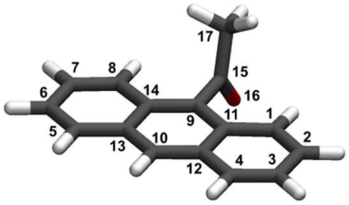

Figure S21. Numbering of positions in 9-acetylanthracene (9AA).

Table S17. Bond distances (B), planar (A) and dihedral (D) angles for the relevant equilibrium geometries of the different low-lying electronic states of 9-acetylanthracene. The terms GP, CHX, CLF, MET, and ACT refer respectively to gas phase, cyclohexane, chloroform, methanol, and acetonitrile. Figure S22 shows the atomic numbering scheme.

Parameter	S_0					^1L_a					^3L_b					$^3n\pi^*$					^3L_a				
	GP	CHX	CLF	MET	ACT	GP	CHX	CLF	MET	ACT	GP	CHX	CLF	MET	ACT	GP	CHX	CLF	MET	ACT	GP	CHX	CLF	MET	ACT
D(11,9,15,16)	82.12	89.69	89.64	90.26	90.2	40.69	40.36	39.47	38.38	38.42	79.82	80.75	81.1	81.03	80.97	12.92	11.87	11.05	10.12	9.98	46.23	48.93	50.8	53.26	53.27
D(1,11,9,15)	-1.93	-1.44	-0.88	-0.56	-0.55	-11.32	-10.27	-9.96	-10.08	-10.06	-1.87	-1.06	-0.47	0.01	0	-13.15	-13.13	-13.22	-13.6	-13.62	-9.23	-6.53	-4.44	-2.7	-2.69
A(9,15,16)	121.4	121.0	120.6	120.4	120.4	121.8	121.7	121.7	121.7	121.7	121.5	121.0	120.7	120.4	120.4	123.3	123.0	122.9	122.7	122.7	121.6	121.3	121.0	120.8	120.8
B(9,15)	1.511	1.510	1.510	1.510	1.510	1.484	1.480	1.476	1.473	1.473	1.509	1.509	1.509	1.508	1.508	1.423	1.426	1.430	1.433	1.432	1.487	1.487	1.487	1.488	1.488
B(15,16)	1.209	1.212	1.214	1.216	1.216	1.225	1.230	1.234	1.238	1.238	1.209	1.212	1.214	1.216	1.216	1.289	1.288	1.287	1.286	1.286	1.219	1.221	1.223	1.224	1.224
B(15,17)	1.508	1.504	1.502	1.500	1.500	1.518	1.516	1.515	1.514	1.514	1.508	1.504	1.502	1.500	1.500	1.522	1.523	1.523	1.523	1.523	1.517	1.514	1.511	1.509	1.509
B(1,2)	1.365	1.365	1.365	1.366	1.366	1.406	1.406	1.407	1.407	1.407	1.408	1.409	1.409	1.410	1.410	1.372	1.372	1.372	1.372	1.372	1.407	1.407	1.408	1.408	1.408
B(2,3)	1.426	1.427	1.427	1.428	1.428	1.387	1.387	1.387	1.387	1.387	1.390	1.390	1.391	1.391	1.391	1.418	1.420	1.421	1.422	1.422	1.383	1.384	1.384	1.384	1.384
B(3,4)	1.363	1.364	1.364	1.365	1.365	1.400	1.400	1.400	1.400	1.400	1.404	1.405	1.405	1.405	1.405	1.366	1.366	1.366	1.366	1.366	1.402	1.403	1.404	1.404	1.404
B(4,12)	1.432	1.433	1.433	1.433	1.433	1.407	1.407	1.407	1.407	1.407	1.448	1.449	1.449	1.449	1.449	1.430	1.431	1.432	1.433	1.433	1.401	1.402	1.402	1.402	1.402
B(11,12)	1.437	1.437	1.437	1.437	1.437	1.440	1.441	1.441	1.441	1.441	1.423	1.423	1.423	1.423	1.423	1.436	1.437	1.438	1.438	1.438	1.434	1.435	1.435	1.435	1.435
B(1,11)	1.434	1.435	1.435	1.435	1.435	1.406	1.405	1.405	1.405	1.405	1.448	1.448	1.449	1.449	1.449	1.420	1.421	1.422	1.423	1.423	1.401	1.401	1.402	1.402	1.402
B(9,11)	1.406	1.406	1.406	1.406	1.406	1.442	1.446	1.449	1.451	1.451	1.408	1.408	1.409	1.409	1.409	1.443	1.442	1.441	1.440	1.440	1.453	1.452	1.452	1.450	1.450
B(10,12)	1.396	1.396	1.397	1.397	1.397	1.411	1.412	1.413	1.413	1.413	1.398	1.398	1.399	1.399	1.399	1.397	1.397	1.396	1.396	1.396	1.424	1.424	1.425	1.425	1.425
B(10,13)	1.396	1.397	1.397	1.397	1.397	1.409	1.410	1.410	1.410	1.410	1.398	1.398	1.399	1.399	1.399	1.400	1.401	1.401	1.401	1.401	1.422	1.423	1.423	1.423	1.423
B(13,14)	1.436	1.437	1.437	1.437	1.437	1.439	1.439	1.439	1.438	1.438	1.422	1.423	1.423	1.423	1.424	1.433	1.434	1.435	1.435	1.435	1.431	1.432	1.433	1.433	1.433
B(9,14)	1.405	1.406	1.406	1.406	1.406	1.442	1.445	1.447	1.450	1.450	1.407	1.408	1.408	1.409	1.409	1.444	1.443	1.442	1.441	1.441	1.452	1.451	1.450	1.448	1.448
B(8,14)	1.434	1.435	1.435	1.435	1.435	1.403	1.402	1.402	1.402	1.402	1.448	1.448	1.448	1.448	1.448	1.424	1.426	1.427	1.428	1.428	1.399	1.400	1.400	1.401	1.401
B(5,13)	1.432	1.433	1.433	1.433	1.433	1.408	1.408	1.408	1.409	1.409	1.447	1.448	1.448	1.449	1.449	1.429	1.430	1.430	1.430	1.430	1.402	1.403	1.403	1.403	1.403
B(5,6)	1.364	1.364	1.364	1.365	1.365	1.398	1.399	1.399	1.399	1.399	1.404	1.404	1.405	1.405	1.405	1.367	1.367	1.368	1.368	1.368	1.401	1.403	1.403	1.404	1.404
B(6,7)	1.426	1.427	1.427	1.428	1.428	1.389	1.389	1.389	1.389	1.389	1.390	1.390	1.391	1.391	1.391	1.416	1.417	1.418	1.418	1.418	1.384	1.385	1.385	1.385	1.385
B(7,8)	1.365	1.365	1.366	1.366	1.366	1.405	1.406	1.406	1.406	1.407	1.408	1.409	1.409	1.409	1.409	1.373	1.374	1.373	1.373	1.373	1.406	1.407	1.408	1.408	1.408

REFERENCES

1) Tamaki, T., Solvent effects on the fluorescence quantum yields and lifetimes of 1-and 2-acetyl-or benzoylanthracenes. *Bull. Chem. Soc. Jpn*. **1982**, 55 (6), 1756-1760.

2) Johnson, A. E.; Jarzęba, W.; Walker, G. C.; Barbara, P. F., Ultrafast Torsional Relaxation from the Barrier Region for an Excited-State Isomerization in Solution: 9-Carbonylanthracenes. *Isr. J. Chem*. **1993**, 33 (2), 199-206.

3) Hirayama, S. Effect of substituent on the behaviour of the excited singlet and triplet states in carbonyl derivatives of anthracene of the type 9-X· CO· A. *Journal of the Chemical Society, Faraday Transactions 1: Physical Chemistry in Condensed Phases*, **1982**, *78*(8), 2411-2421.

4) Zobel, J. P., Nogueira, J. J., González, L. Quenching of charge transfer in nitrobenzene induced by vibrational motion. *J. Phys. Chem. Letters*, **2015**, *6*(15), 3006-3011.

5) Zobel, J. P., Nogueira, J. J., González, L. Mechanism of Ultrafast Intersystem Crossing in 2-Nitronaphthalene. *Chemistry–A European Journal*, **2018**, *24*(20), 5379-5387.

6) Mai, S., Marquetand, P., González, L. Nonadiabatic dynamics: The SHARC approach. *Wiley Interdisciplinary Reviews: Computational Molecular Science*, **2018**, *8*(6), e1370.

7) De Souza, B., Neese, F., Izsák, R. (2018). On the theoretical prediction of fluorescence rates from first principles using the path integral approach. *Journal of Chemical Physics*, **2018**, *148*(3) 034104.

8) Matsumoto, T., Sato, M., Hirayama, S. Intersystem crossing in 9-carbonyl derivatives of anthracene. *Chem. Phys. Lett.* **1972**, *13*(1), 13-15.
